# Murine Butyrophilin-Like 1 and Btnl6 Form Heteromeric Complexes in Small Intestinal Epithelial Cells and Promote Proliferation of Local T Lymphocytes

**DOI:** 10.3389/fimmu.2016.00001

**Published:** 2016-01-19

**Authors:** Cristina Lebrero-Fernández, Joakim H. Bergström, Thaher Pelaseyed, Anna Bas-Forsberg

**Affiliations:** ^1^Department of Microbiology and Immunology, Institute of Biomedicine, University of Gothenburg, Gothenburg, Sweden; ^2^Department of Medical Biochemistry and Cell Biology, Institute of Biomedicine, University of Gothenburg, Gothenburg, Sweden

**Keywords:** butyrophilin-like, intraepithelial lymphocytes, mucosal immunity, intestinal epithelial cells, γδ T cells

## Abstract

To date, few molecular conduits mediating the cross-talk between intestinal epithelial cells and intraepithelial lymphocytes (IELs) have been described. We recently showed that butyrophilin-like (Btnl) 1 can attenuate the epithelial response to activated IELs, resulting in reduced production of proinflammatory mediators, such as IL-6 and CXCL1. We here report that like Btnl1, murine Btnl6 expression is primarily confined to the intestinal epithelium. Although Btnl1 can exist in a cell surface-expressed homomeric form, we found that it additionally forms heteromeric complexes with Btnl6, and that the engagement of Btnl1 is a prerequisite for surface expression of Btnl6 on intestinal epithelial cells. In an IEL-epithelial cell coculture system, enforced epithelial cell expression of Btnl1 significantly enhanced the proliferation of IELs in the absence of exogenous activation. The effect on proliferation was dependent on the presence of IL-2 or IL-15 and restricted to IELs upregulating CD25. In the γδ T-cell subset, the Btnl1–Btnl6 complex, but not Btnl1, specifically elevated the proliferation of IELs bearing the Vγ7Vδ4 receptor. Thus, our results show that murine epithelial cell-specific Btnl proteins can form intrafamily heterocomplexes and suggest that the interaction between Btnl proteins and IELs regulates the expansion of IELs in the intestinal mucosa.

## Introduction

The butyrophilin (Btn)- and the butyrophilin-like (Btnl) genes have over recent years emerged as potent immune regulators and have attracted increasing attention by immunologists. The Btn and Btnl genes belong to the immunoglobulin (Ig) superfamily and have structural resemblance to the B7 family genes, which encode positive (e.g., B7-1, B7-2, and ICOS) and negative (e.g., PD-L1, PD-L2, B7-H3, and B7-H4) regulators of T lymphocytes. Like the B7 proteins, several of both human and murine Btn and Btnl members have been reported to control the biological activity of peripheral T cells by regulating their activation and proliferation by anti-CD3 (1–7). In addition, human Btnl2 has been genetically associated with a growing number of inflammatory conditions, such as sarcoidosis, myositis, rheumatoid arthritis, and inflammatory bowel disease (8–11). These diseases are characterized by inappropriate T cell activation (12, 13), and thus, the genetic linkage further suggests the family’s implication in T cell regulation and inflammation. Moreover, human BTN2A1 has been shown to modulate immature dendritic cells (DC) by binding to the dendritic cell-specific intercellular adhesion molecule-3-grabbing non-integrin (DC-SIGN) (14), and Btnl2 has been found to induce Foxp3 expression in T cells, thus promoting the development of regulatory T lymphocytes (15). Furthermore, it was recently reported that human BTN3A1 can present phosphoantigens to human Vγ9Vδ2 T cells, and hence can act as an antigen-presenting molecule regulating the function of unconventional T cells (16, 17). Taken together, it has become increasingly evident that the Btn and Btnl genes govern pleiotropic functions implicated in balancing the immune system. Although most studies have focused on defining the role of the Btn and Btnl family in immune responses in the periphery, few studies have considered the implication of gene expression for local immune responses in the tissue. We have previously reported Btnl1-specific regulation of intraepithelial lymphocyte (IEL)–epithelial cell interactions in the murine small intestinal mucosa, and shown that Btnl1 is involved in suppressing proinflammatory mediators of the NFκB pathway, such as IL-6, IL-15, CXCL1, and CCL4 (18). To further identify the role of Btnl genes in regulating local gut immune responses, we continued the characterization of Btnl1 and additionally examined the role of Btnl6, one of Btnl1’s closest relatives, by defining its protein expression pattern, identifying its biological form, and characterizing its potential to regulate intestinal IELs.

## Materials and Methods

### Mice

Six-week-old C57BL/6J and C3H/HeN mice were purchased from Harlan Laboratories (Netherlands) and Janvier Labs, respectively, and maintained in the Laboratory of Experimental Biomedicine, Gothenburg University (Gothenburg, Sweden). Protocols were approved by the government animal ethics committee (permit no. 335-2012), and institutional animal use and care guidelines were followed.

### Cell Lines

Murine intestinal epithelial cell line MODE-K [derived from C3H/He mice (19)], human embryonic kidney (HEK) 293 cell line, and murine fibroblast cell line 3T3 were maintained at 37°C, 5% CO_2_ in Dulbecco’s modified essential medium (DMEM; Gibco^®^, Life Technologies) plus 10% FCS (PAA Laboratories), 100 U/ml penicillin, 100 μg/ml streptomycin, 0.292 mg/ml glutamine, and 1× non-essential amino acids (Gibco^®^, Life Technologies).

### Generation of Transiently Transfected HEK 293- and MODE-K Cells

Human embryonic kidney 293 cells and MODE-K cells were transfected with Btnl1-, Btnl4-, Btnl6-, Btnl4 + Btnl1-pMX-IRES-GFP, Btnl6 + Btnl1-pMX-IRES-GFP, or pMX-IRES-GFP using polyethylenimine (PEI; Polysciences, Warrington, PA, USA) or lipofectamine (Invitrogen™, Life Technologies) according to standard procedures.

### Generation of Stably Transfected N-Terminal FLAG-Tagged Btnl-pMX-IRES-GFP-MODE-K Cells

Stably transfected MODE-K cells were generated, as previously described (18).

### Generation of Btnl6 Polyclonal Antibody

A KLH-conjugated synthetic peptide derived from the extracellular murine Btnl6 protein sequence was injected into New Zealand White rabbits. Preimmune serum was collected from each rabbit as a negative control. Antisera were collected post immunization, and reactivity tested by ELISA against the original peptide. The antibody was purified on a peptide column, whereas preimmune sera were purified over protein A.

### Isolation and Culture of Murine Small Intestinal IELs

Intraepithelial lymphocytes were isolated from mouse small intestine, as previously described (18, 20, 21). The isolated IELs were cultured in the presence of 1 μg/ml anti-CD3ɛ (clone 145-2C11, BD Pharmingen) and a cytokine cocktail containing IL-2 (10 U/ml) (Roche), IL-3 (100 U/ml) (R&D), IL-4 (200 U/ml) (R&D), and IL-15 (100 ng/ml) (R&D) for 48 h and thereafter transferred to fresh wells and cultured only in the presence of IL-2 (10 U/ml). Cells were maintained in 96-well round-bottom plates at 37°C and 10% CO_2_. Medium was replaced every 3–4 days.

### Flow Cytometric Analysis

Btnl-transfected cells were stained with APC-conjugated rabbit anti-FLAG or anti-HA antibody (PerkinElmer). For detection of intracellular expression of FLAG-Btnl6, cells were permeabilized using the cytofix/cytoperm kit (BD Biosciences). CFSE-labeled cells were stained with Alexa Fluor 700-conjugated anti-CD45 (eBioscience), eFluor450-conjugated anti-pan TCRγδ (GL3, eBioscience), APC or APC-Cy7-conjugated anti-TCRb (eBioscience), PerCPCy5.5-conjugated anti-CD25 (eBioscience), PE-conjugated anti-TCR Vγ1.1/Cr4 (BioLegend), eFluor660-conjugated anti-TCR Vδ4 (eBioscience), anti-TCR Vγ7 (kindly provided by Dr. Pablo Pereira, Institut Pasteur, Paris, France), and 7-aminoactinomycin D (7AAD; Sigma-Aldrich) or LIVE/DEAD^®^ Fixable Red Dead Cell Stain (Molecular Probes^®^, Life Technologies). Cells were gated on 7AAD or LIVE/DEAD^®^ Fixable Red negative cells to exclude non-viable cells. Cells were acquired on LSR II flow cytometer using the DIVA software (BD Biosciences), and analysis of data was performed using the FlowJo Software version 7.6.5.

### Confocal Microscopy

MODE-K cells were plated on collagen-coated coverslips 1 day prior to transfection. On day 2, cells were transfected using Lipofectamine (Invitrogen™, Life Technologies) according to the standard procedures and left to expand in a 37°C 5% CO_2_ incubator for 24 h. On the day of staining, cells were incubated with 10% normal horse serum (NHS), and thereafter with a rabbit anti-HA antibody (Sigma-Aldrich) and a goat anti-rabbit Cy5 (Jackson ImmunoResearch). Cells were blocked with NHS, stained with PE-conjugated anti-FLAG (Prozyme), fixed in 4% paraformaldehyde, and stained with 4′,6-diamidino-2-phenylindole (DAPI). Cells were viewed using confocal microscopy (Zeiss LSM700 Inverted) and analyzed with ZEN lite 2011 microscope software (Carl Zeiss).

### Western Blotting

Tissues, harvested from 6- to 9-week-old female C57BL/6 mice, were homogenized in cell lysis buffer (50 mM Tris, pH 8, 150 mM NaCl, 1% Triton X-100) containing complete protease inhibitors cocktail tablets (Roche Diagnostics). Isolated small intestinal epithelial cells, lamina propria lymphocytes (LPLs), and IELs were lysed in cell lysis buffer. Cell and tissue lysates were clarified by centrifugation, and protein concentration was measured with BCA Protein Assay Kit (Pierce). Ten micrograms of protein were denaturated in reducing sample buffer (NuPAGE LDS 4×; Novex^®^, Life Technologies) containing 1M DTT (Sigma-Aldrich) and loaded onto a NuPage 4–12% Bis-Tris Gel (Novex^®^, Life Technologies). Separated proteins were transferred onto nitrocellulose transfer membranes (Millipore) that were immunoblotted using anti-FLAG antibody (Sigma-Aldrich), anti-GFP antibody (Sigma-Aldrich), anti-Btnl6 rabbit polyclonal antiserum (Moravian-Biotech), rabbit preimmune serum, or anti β-actin antibody (Sigma-Aldrich), and detected with HRP-conjugated goat anti-mouse antibody or HRP-conjugated goat anti-rabbit antibody (Jackson ImmunoResearch).

### Cell Surface Biotinylation and Immunoprecipitation

Cell surface proteins were biotinylated using EZ-Link Sulfo-NHS-LC-Biotin (Thermo Scientific). Briefly, attached cells were washed with phosphate-buffered saline (PBS), pH 8.0. Cells were incubated for 1 h at room temperature with 2 mM of the biotinylation reagent in PBS, pH 8.0. The biotinylation reaction was quenched by washes of PBS, pH 7.4, and 100 mM Glycine. Upon quenching, cells were lysed in 25 mM Tris–HCl pH 7.4, 150 mM NaCl, 1 mM EDTA, 1% NP-40, and 5% glycerol, homogenized, and centrifuged. Immunoprecipitations were performed using FLAG M2 monoclonal antibody (Sigma-Aldrich), cross-linked to magnetic Dynabeads Protein G (Novex^®^, Life Technologies) using 3.5 mg/ml dimethyl pimelimidate (DMP; Sigma-Aldrich) followed by quenching in 25 mM ethanolamine in PBS and removal of excess antibody by 1M glycine pH 3. Cell lysates were incubated overnight at 4°C with beads cross-linked with FLAG M2 antibody, followed by three washes with lysis buffer prior to elution of bound material using 1M glycine pH 3. Eluted material was boiled in reducing sample buffer at 95°C for 5 min. Alternatively, cell lysates were incubated with anti-HA polyclonal antibody (Sigma-Aldrich) overnight at 4°C. Next, Protein G Plus-Agarose (Santa Cruz) was added, and samples were incubated for additional 2 h at 4°C before beads were washed with lysis buffer and boiled in reducing sample buffer at 95°C for 5 min.

### Isolation of Murine Small Intestinal Epithelial Cells

Murine epithelial cells were isolated from small intestinal tissue, as previously described (22).

### Immunoprecipitation and Tandem Mass Spectrometric Analysis (nanoLC-MS/MS)

Purified mouse small intestinal epithelial cells were lysed in extraction buffer with the addition of 100 mM NaCl and protease-inhibitor mixture (Roche) according to Dynabeads Co-Immunoprecipitation Kit protocol (Life Technologies), and clarified by centrifugation. Immunoprecipitation was performed using anti-Btnl1 antibody or preimmune rabbit serum cross-linked to magnetic Epoxy Dynabeads (Life Technologies). Five milligrams of antibody-coated beads were incubated with lysate from 1 × 10^7^ small intestinal epithelial cells. Bound proteins were eluted after washes in alkaline conditions (pH = ~11) using 0.5M NH_4_OH with 0.5 mM EDTA. Eluates were lyophilized and resuspended in non-reducing SDS-PAGE loading buffer and separated on 6% SDS-PAGE gel. Gels were blotted by semidry blot to Immobilon PSQ membranes (Millipore) or stained by Coomassie with Imperial stain (Thermo Scientific). From the Imperial-stained gel, a total of four bands were selected and excised. The proteins were in-gel digested with trypsin (Promega), and the eluted peptides were analyzed by nanoflow liquid chromatography tandem mass spectrometry (nLC-MS/MS) using an Easy-nLC 1000 system (Thermo) coupled to a Q-Exactive mass spectrometer (Thermo) through a nanoelectrospray ion source. Peptides were separated with reverse-phase column (150 mm × 0.075 mm inner diameter, C18-AQ 3 μm) by a 60-min gradient. Full mass spectra were acquired from 350 to 1600 *m*/*z* with resolution of 70,000 (*m*/*z* 200). Up to 12 most intense peaks (charge state ≥2) were fragmented and tandem mass spectrum was acquired with a resolution of 35,000 and dynamic exclusion 30 s. The tandem mass spectral data produced were searched against the *Mus musculus* NCBI database downloaded 29-May-2015 using the Mascot search program (Matrix Science) with search parameters set to: MS accuracy 5 ppm, MS/MS accuracy 0.5 Da, trypsin digestion with one missed cleavage allowed, and variable modifications were set for carbamidomethyl (C), propionamide (C), oxidation (M), and acetylation (protein N-terminal).

### *In Vitro* T Cell Proliferation Assay

Prior to coculture with IELs, MODE-K cells transfected with N-FLAG-Btnl6-pMX-IRES-GFP + N-HA-Btnl1-pMX-IRES-GFP, N-FLAG-Btnl1-pMX-IRES-GFP, or pMX-IRES-GFP were plated on 48- or 24-well flat-bottom tissue culture plates uncoated or precoated with 1 μg/ml anti-CD3ɛ (clone 145-2C11, BD Pharmingen). The following day, when the MODE-K monolayers were ~70% confluent, the medium was replaced with supplemented RPMI 1640 with or without IL-2 (10 U/ml) or IL-15 (50 ng/ml), to which CFSE (Molecular Probes^®^, Life Technologies) labeled IELs were added at 1 × 10^5^ cells/well. IELs were left to proliferate for 72 or 96 h and were thereafter stained with anti-CD45 to exclude GFP^+^ MODE-K cells. Cells were gated on LIVE/DEAD^®^ Fixable Red (Molecular Probes^®^, Life Technologies) negative cells to exclude non-viable cells.

Splenocytes from C57BL/6 mice were depleted of B-cells by negative selection with anti-CD19 microbeads (Miltenyi Biotec) using an auto-MACS separator. The purity of cells was analyzed by flow cytometry and was >95% in all experiments performed. Splenocytes were labeled with CFSE and were stimulated with anti-CD3ɛ (clone 145-2C11, BD Pharmingen) and anti-CD28 (clone 37.51, BD Pharmingen) in the presence of Btnl1-, Btnl1 + 6, or pMX transfected MODE-K cells. Proliferative response was assessed by flow cytometry after staining with anti-CD45 to exclude GFP^+^ MODE-K cells, and after gating on LIVE/DEAD^®^ Fixable Red (Molecular Probes^®^, Life Technologies) negative cells to exclude non-viable cells.

### Cytokine Measurement in Cell Culture Supernatant

Culture supernatants were analyzed by flow cytometry using Mouse Th1/Th2/Th17/Th22 13plex Kit FlowCytomix (eBioscience) according to the manufacturer’s instructions. The samples were acquired in LSR II flow cytometer. Analysis of data and quantification of cytokines was performed using the FlowCytomix Pro Software (eBioscience) on the basis of corresponding standards curves.

### Statistical Analysis

All data were generated using GraphPad Prism version 6.04. Significance between conditions was determined by unpaired two-tailed *t*-test. Differences were considered as statistically significant when *P* < 0.05 (*P ≤ 0.05, **P ≤ 0.01, ***P ≤ 0.001, and ****P ≤ 0.0001). Correlation between CD25 expression and IEL proliferation was determined using Spearman correlation test.

## Results

### Btnl6 Protein Expression

**Figure 1:**
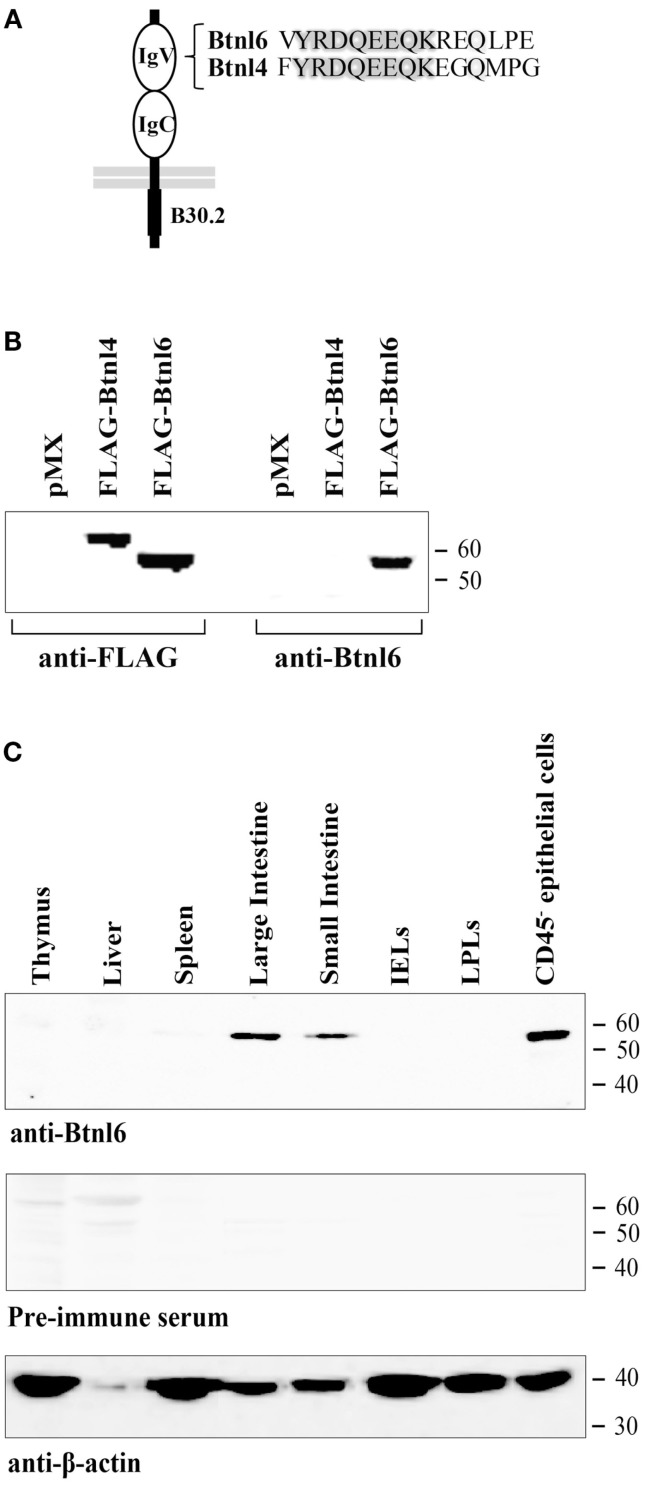
**Validation of the Btnl6 antibody and Btnl6 protein expression in tissue**. **(A)** A rabbit anti-mouse Btnl6-reactive antibody was developed against a synthetic peptide (residues 79–93). Peptide alignment across Btnl4 and 6 is shown. **(B)** Lysates from HEK 293 cells transfected with FLAG-tagged Btnl4 cDNA pMX-IRES-GFP, FLAG-tagged Btnl6 cDNA pMX-IRES-GFP, or empty vector (pMX-IRES-GFP) were divided and immnoblotted either using anti-FLAG- or anti-Btnl6 antibody. A single band consistent with the theoretical molecular weight of ~64 kDa for FLAG-tagged Btnl4 and ~59 kDa for FLAG-tagged Btnl6 was detected under reducing conditions when immunoblotted with anti-FLAG. Anti-Btnl6 antibody detected Btnl6 transfectants, but not Btnl4 transfectants or HEK 293 cells transfected with empty vector. **(C)** Tissues from C57BL/6 mice were analyzed for Btnl6 protein expression using a Btnl6-specific polyclonal antibody. A single band consistent with the theoretical molecular weight of ~58 kDa for Btnl6 was detected in large and small intestine, and in small intestinal epithelial cells under reducing conditions. No bands were detected on gels immunoblotted with preimmune serum. The β-actin immunoblot acts as a loading control. IELs, intraepithelial lymphocytes; LPLs, lamina propria lymphocytes. Data are representative of two experiments.

To study the expression of Btnl6 protein, a rabbit polyclonal antibody was generated to a peptide from the putative IgV region that was 66% conserved with the Btnl4 protein, a Btnl-family member most homologous to Btnl6 (Figure 1A) (23). To confirm the specificity of the antibody, lysates from HEK 293 cells transfected with Btnl4 or Btnl6 cDNA that included a N-terminal FLAG epitope C-terminal to the putative signal cleavage site were divided and immunoblotted either using an anti-FLAG or anti-Btnl6 antibody. The predicted proteins, migrating on reducing gels at the theoretical molecular weight of ~64 kDa for FLAG-tagged Btnl4 and ~59 kDa for FLAG-tagged Btnl6, were detected with anti-FLAG. Anti-Btnl6 antibody detected Btnl6 transfectants, but did not detect Btnl4 transfectants or HEK 293 cells transfected with empty vector (Figure 1B).

We used the anti-Btnl6 antibody to screen a panel of mouse tissues previously shown to express Btnl6 transcripts (18) for Btnl6 protein expression by Western blotting. Btnl6 expression was readily observed, migrating at ~58 kDa (the predicted molecular mass for non-tagged Btnl6), in small and large intestine. The expression in the intestine was confined to epithelial CD45^−^ cells (Figure 1C) and was not detected in IEL nor LPL lysates, which was consistent with the previously published mRNA analysis (18).

### Btnl1 Promotes Surface Expression of Btnl6 on Small Intestinal Epithelial Cells

**Figure 2:**
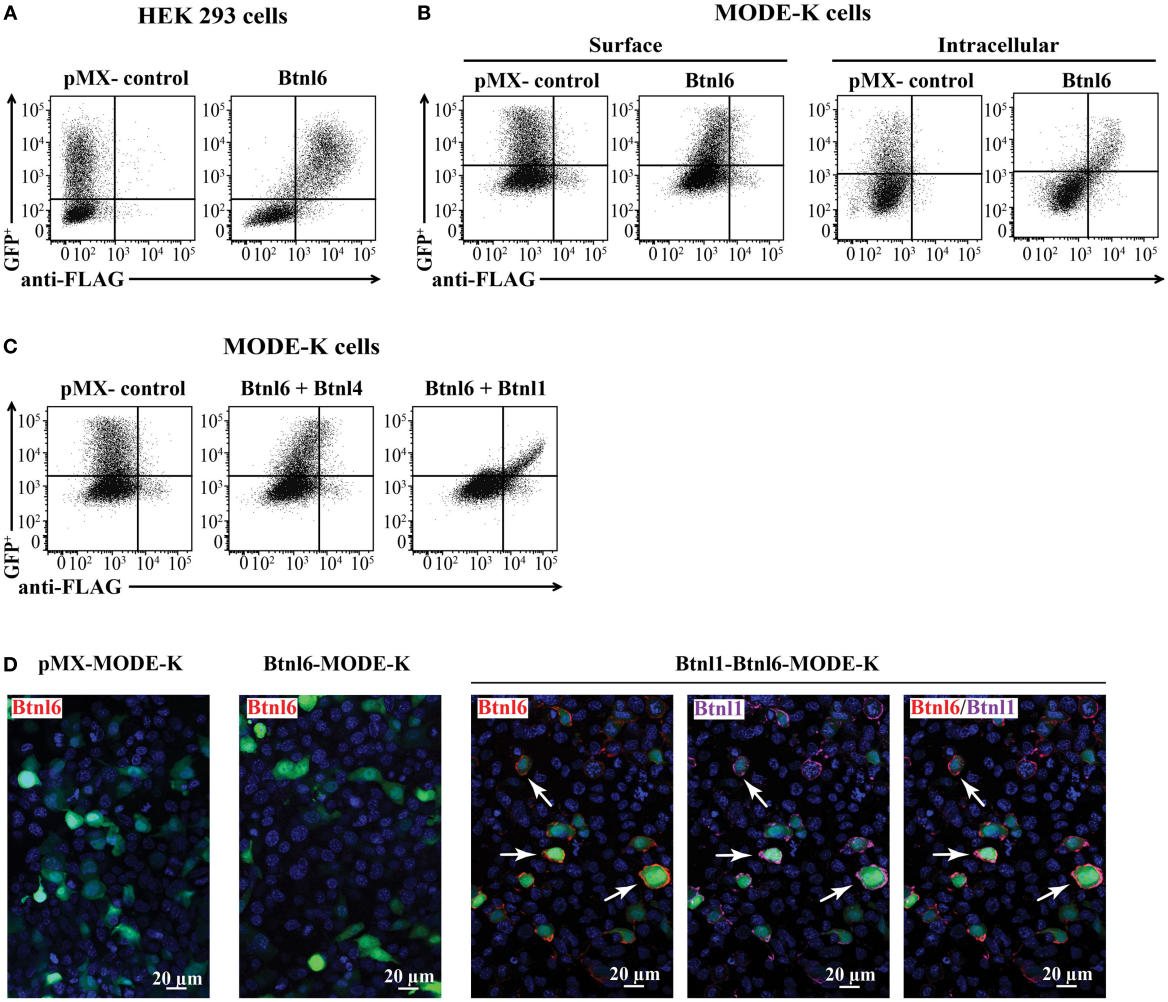
**Btnl6 expression**. **(A)** HEK 293 cells were transfected with N-terminal FLAG-tagged Btnl6 cDNA pMX-IRES-GFP. An anti-FLAG antibody detected cell surface expression of Btnl6 but not gfp^+^ HEK 293 cells transfected with empty vector (pMX-IRES-GFP). **(B)** Murine small intestinal epithelial cells, MODE-K cells, were transfected with N-terminal FLAG-tagged Btnl6 cDNA pMX-IRES-GFP. Cells were stained with an anti-FLAG antibody for cell surface expression of Btnl6, or permeabilized for detection of intracellular Btnl6 localization. Btnl6 could not be detected on the plasma membrane of transfected MODE-K cells. Instead, Btnl6 was retained in the intracellular compartment as evident by anti-FLAG staining. **(C)** MODE-K cells were cotransfected with untagged Btnl4 cDNA- and N-terminal FLAG-tagged Btnl6 cDNA pMX-IRES-GFP, or untagged Btnl1 cDNA- and N-terminal FLAG-tagged Btnl6 cDNA pMX-IRES-GFP. An anti-FLAG antibody detected cell surface expression of Btnl6 on the Btnl1–Btnl6 cotransfectants, but not on the Btnl4–Btnl6 cotransfectants. Representative plots **(A–C)** from single experiments out of three independent experiments are shown. **(D)** MODE-K cells were transfected with N-terminal FLAG-tagged Btnl6 cDNA pMX-IRES-GFP, FLAG-tagged Btnl6 cDNA- and HA-tagged Btnl1 cDNA pMX-IRES-GFP, or empty vector (pMX-IRES-GFP). While no Btnl6 expression was detected on the cell surface of MODE-K cells transfected with Btnl6, Btnl6 was detected on most Btnl1–Btnl6 cotransfectants. Btnl6-, Btnl1-, and double-stained Btnl1 + Btnl6 expressing cells are exemplified by arrows. Anti-FLAG antibody was used for detection of FLAG-Btnl6 (red) and anti-HA antibody was used for detection of HA-Btnl1 (purple). Slides were counterstained with DAPI (blue) to visualize nuclei. The green staining indicates transfected, GFP^+^ cells. Original magnification 20×. Images are representative of three experiments.

The Btnl6 gene is predicted to encode a transmembrane protein with two ectodomains (IgV + C) and a cytoplasmic B30.2 domain. To characterize the expression of Btnl6, a panel of cell lines was transfected with N-terminal-FLAG-tagged Btnl6 cDNA cloned into a bicistronic pMX-IRES-GFP expression vector. Although Btnl6 was readily displayed on the cell surface of the heterologous HEK 293 cells (Figure 2A), and the murine 3T3 fibroblast cell line (data not shown), Btnl6 was not detected on the plasma membrane of the physiologically relevant MODE-K cells, which represent small intestinal epithelial cells (Figure 2B) (none of the cell lines ordinarily express Btnl proteins). Instead, in these cells, the protein was retained in the intracellular compartment (Figure 2B). The mode of Btnl6 expression thus differs from Btnl1, which upon transfection localizes to the plasma membrane of MODE-K cells (18). In an attempt to induce Btnl6 cell surface expression, Btnl6-transfected MODE-K cells were incubated with activated IEL supernatants, a treatment previously shown to upregulate surface Btnl1 on enterocytes (18). Whereas activated IEL supernatants failed to induce Btnl6 expression (data not shown), cotransfection with cDNA encoding the Btnl1 protein induced Btnl6 on the cell surface of MODE-K cells (Figure 2C). This effect was specific for Btnl1 as Btnl6 did not localize to the plasma membrane upon cotransfection of MODE-K cells with Btnl4 cDNA (Figure 2C), another Btnl family member with reported expression in intestinal epithelial cells (18).

To further characterize the Btnl1-dependent expression of Btnl6, MODE-K cells were plated on cover slips and transfected with FLAG-tagged Btnl6-GFP cDNA, or cotransfected with FLAG-tagged Btnl6-GFP cDNA and N-terminal-HA-tagged Btnl1-GFP cDNA, immunostained with anti-FLAG antibody and analyzed with confocal microscopy. While expression of Btnl6 was readily detected on the plasma membrane of Btnl6–Btnl1 cotransfected GFP^+^ cells, no FLAG positive staining was detected on cells transfected with Btnl6 only (Figure 2D).

### Btnl6 Forms a Heteromeric Plasma Membrane-Tethered Complex with Btnl1 on MODE-K Epithelial Cells

**Figure 3:**
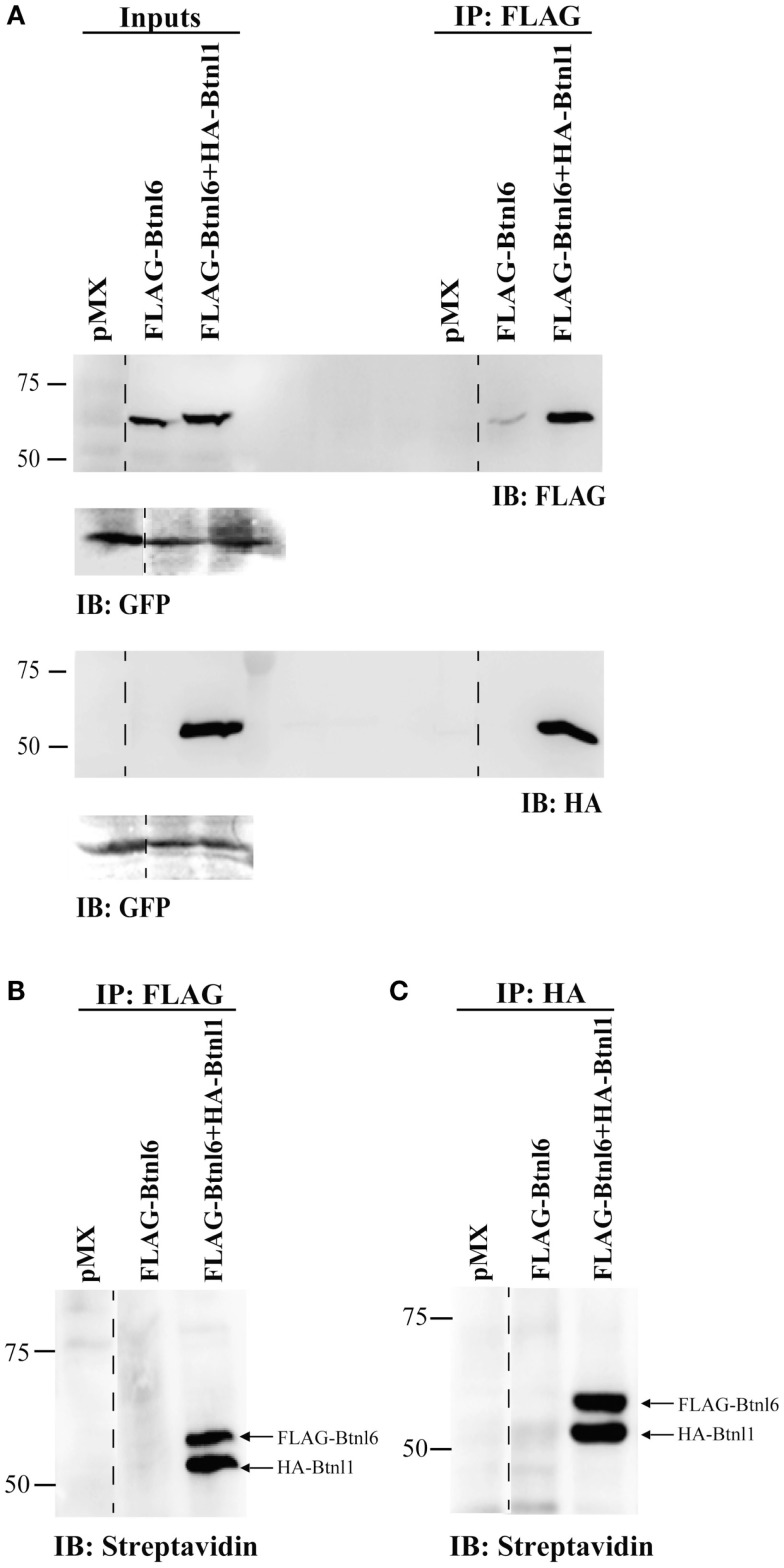
**Btnl6 forms plasma membrane-tethered heteromeric complexes with Btnl1 in small intestinal epithelial cells**. MODE-K cells were transfected with FLAG-tagged Btnl6 cDNA pMX-IRES-GFP, FLAG-tagged Btnl6 cDNA- and HA-tagged Btnl1 cDNA pMX-IRES-GFP, or empty vector (pMX-IRES-GFP). Cell surface proteins were biotinylated prior to cell lysis, and coimmunoprecipitated using FLAG or HA tags. Biotinylated material, immunoprecipitated with anti-FLAG or anti-HA, was either immunoblotted using anti-FLAG or anti-HA **(A)**, or detected with streptavidin-HRP **(B,C)**. GFP immunoblot acts as a loading control. The lanes were reorganized (dashed lines) from the same gel without any image manipulation. Data are representative of two experiments.

To determine if the Btnl1-facilitated surface expression of Btnl6 was mediated by Btnl1–Btnl6 interaction, MODE-K cells were transfected with FLAG-tagged Btnl6-GFP cDNA, or FLAG-tagged Btnl6-GFP cDNA and HA-tagged Btnl1-GFP cDNA. Cell surface proteins were biotinylated with non-cleavable EZ-Link Sulfo NHS-LC-Biotin prior to cell lysis and immunoprecipitation using either FLAG or HA antibody immobilized on magnetic or agarose beads. The biotinylated material, immunoprecipitated with anti-FLAG or anti-HA, was immunoblotted using anti-FLAG antibody or anti-HA antibody, or detected with streptavidin. As expected, anti-FLAG immunoprecipitated FLAG-Btnl6 migrated at ~59 kDa. In lysates cotransfected with FLAG-Btnl6 and HA-Btnl1, anti-FLAG coimmunoprecipitated HA-Btnl1 migrated at ~53 kDa (the predicted molecular mass for HA-tagged Btnl1) as evident from the anti-HA immunoblotting (Figure 3A). In a reciprocal manner, in lysates cotransfected with FLAG-Btnl6 and HA-Btnl1 cDNA, anti-HA coimmunoprecipitated FLAG-Btnl6 (data not shown). These results strongly suggested a Btnl1–Btnl6 interaction, and hence formation of a Btnl1–Btnl6 heterocomplex. In addition, the experiment verified that the complex is plasma membrane localized as both Btnl6- and Btnl1-specific bands were detected after biotinylation of surface-expressed proteins and subsequent immunoprecipitation and immunoblotting with streptavidin (Figures 3B,C). Although Btnl6 was detected in input lanes and in whole cell lysates immunoprecipitated and immunoblotted with anti-FLAG, no Btnl6-specific band was detected after immunoblotting with streptavidin in absence of HA-Btnl1. Hence, surface expression of Btnl6 is regulated by Btnl1 and is facilitated by Btnl1–Btnl6 interaction.

### Btnl6 Heteromerizes with Btnl1 to Form High-Molecular Mass Intrafamily Complexes in Small Intestinal Epithelial Cells

**Figure 4:**
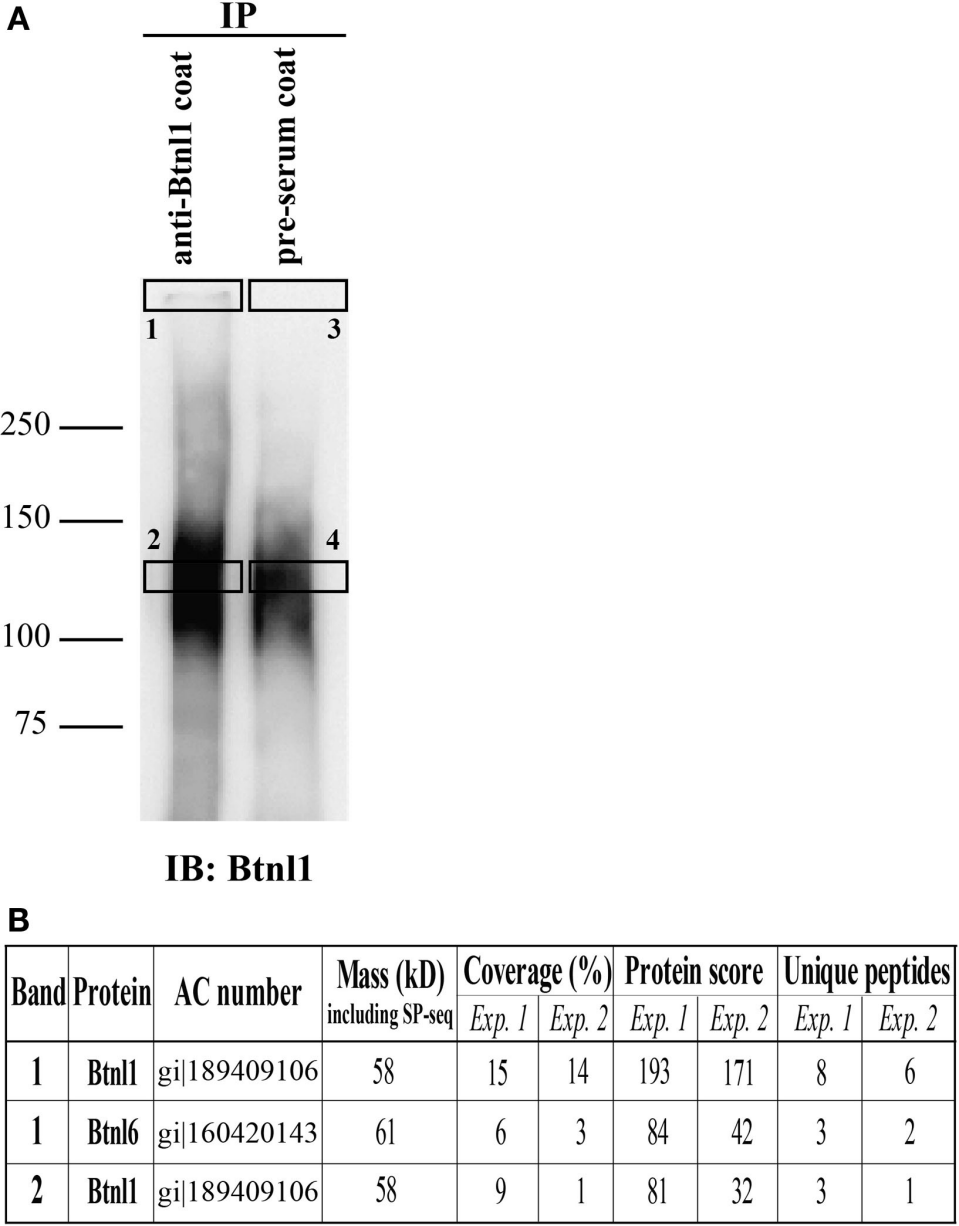
**Btnl1 and Btnl6 form heteromeric interactions in primary small intestinal epithelial cells**. Lysate from isolated primary intestinal epithelial cells were immunoprecipitated with anti-Btnl1 or preimmune rabbit serum-coated beads. Bound protein was released from the beads using alkaline conditions and separated by SDS-PAGE under non-reducing conditions. **(A)** Separated eluates after immunoblot with anti-Btnl1 antibody followed by HRP-conjugated anti-rabbit antibody. The unspecific band in the preimmune serum lane indicates that some bead-coupled antibodies were released in the elution step. Squares (1–4) indicate excised bands on a corresponding Coomassie stained gel subjected to in-gel digestion by trypsin followed by mass spectrometry analysis. **(B)** The table shows identified butyrophilin-like proteins in corresponding excised bands in the two independent experiments performed. Neither Btnl1 nor Btnl6 was detected in samples immunoprecipitated with preimmune serum (band 3 and 4). Data are representative of two independent experiments. AC, accession number; SP, signal peptide. A complete list of identified proteins in the excised gel bands is given in Table S1 in Supplementary Material.

In order to monitor the status of endogenous Btnl6 protein, we used a rabbit polyclonal anti-Btnl1 antibody (18) for protein complex detection in lysates of freshly isolated small intestinal epithelial cells. Cell lysates were subjected to immunoprecipitation using the anti-Btnl1 antibody or preimmune rabbit serum and separated on SDS-PAGE gel, followed by immunoblotting with anti-Btnl1 antibody for complex visualization, or Coomassie staining for mass spectrometry analysis. A non-reduced high-molecular mass complex was detected in the interface between the stacking gel and the separation gel (Figure 4A). The size of the complex was not possible to determine due to the limited migration in the separation gel. Position of the same band was extrapolated on the Coomassie stained gel allowing excision and trypsin digestion of the corresponding proteins. The material was subjected to mass spectrometry which identified Btnl1 and also Btnl6 in the high-molecular mass complex band (Figure 4B). Additionally, anti-Btnl1 also detected a non-reduced complex of ~130 kDa that showed to contain Btnl1, but not other Btnl-family members, suggesting that the complex may include Btnl1 homodimers. Neither Btnl1 nor Btnl6 was detected in samples immunoprecipitated with preimmune serum showing that the identifications are not due to unspecific interactions with the beads. A complete list of identified proteins in the excised gel bands is given in Table S1 in Supplementary Material. Hence, Btnl6 heteromerizes with Btnl1 to form high-molecular mass complexes in small intestinal epithelial cells.

### Btnl1 and Btnl1–Btnl6 Regulate IEL Proliferation in the Absence of Exogenous Activation

**Figure 5:**
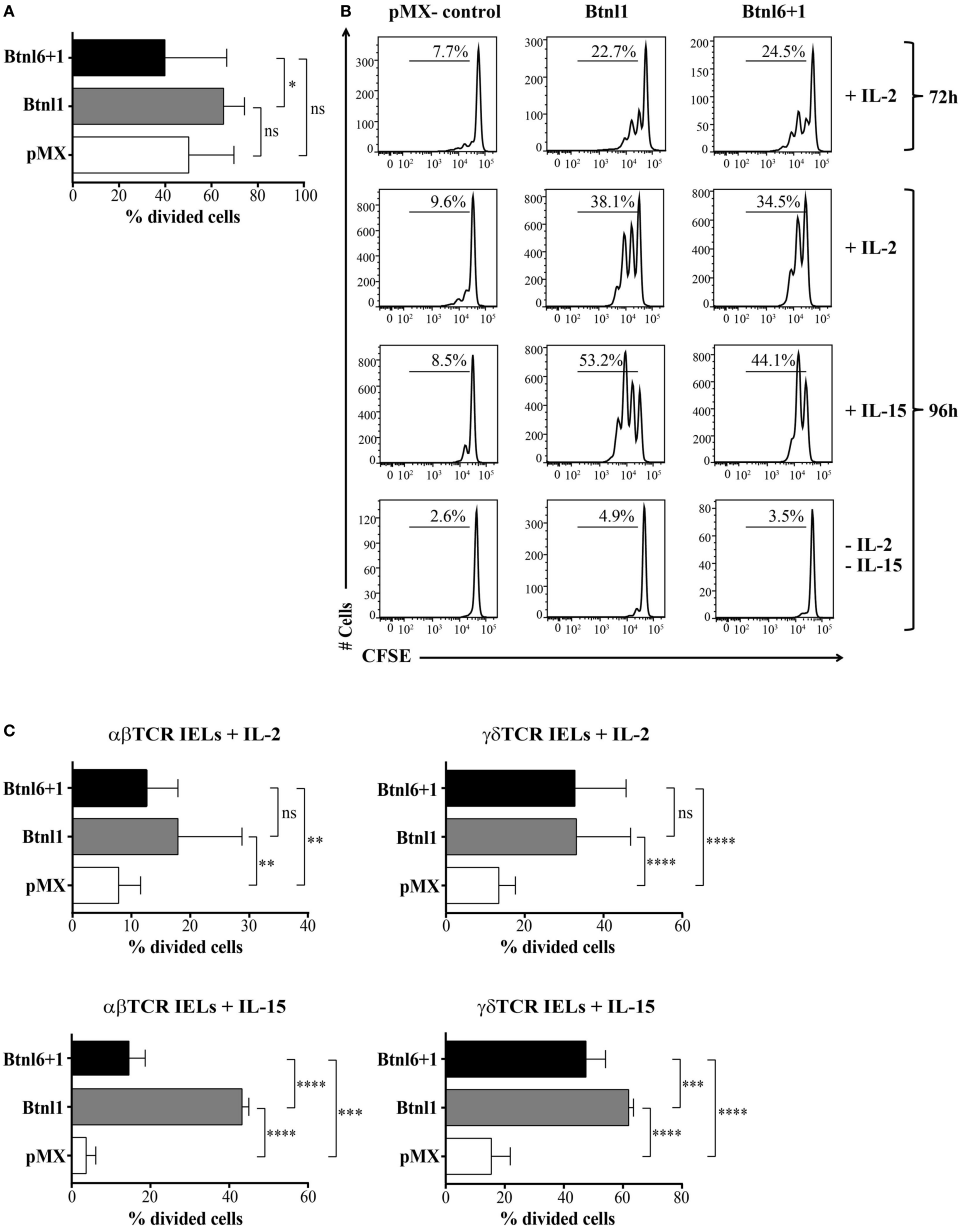
**Btnl proteins regulate IEL proliferation**. **(A)** MODE-K cells transfected with Btnl6 and Btnl1 cDNA pMX-IRES-GFP, Btnl1 cDNA pMX-IRES-GFP, or empty vector (pMX-IRES-GFP) were cocultured with CFSE-labeled IELs in the presence of anti-CD3 activation. IELs were left to proliferate, and the cell division was monitored after 96 h. Graphs show the mean ± SD, and data are pooled from five independent experiments, each performed in duplicates. **(B,C)** MODE-K cells transfected with Btnl6 and Btnl1 cDNA pMX-IRES-GFP, Btnl1 cDNA pMX-IRES-GFP, or empty vector (pMX-IRES-GFP) were cocultured with CFSE-labeled IELs in the absence of anti-CD3 activation, with or without IL-2 or IL-15. IELs were left to proliferate, and cell division was monitored after 72 and 96 h **(B)** or 96 h **(C)**. Proliferation was assessed on total IELs **(B)** or IELs gated on αβ TCR or γδ TCR IEL subsets **(C)**. Representative histograms from single experiments out of seven independent experiments for IL-2, each performed in duplicates, and out of two independent experiments for IL-15, each performed in triplicates, are shown. Graphs show the mean ± SD. Proliferative response was assessed by flow cytometry after staining with anti-CD45 to exclude GFP^+^ MODE-K cells, and after gating on LIVE/DEAD *Fixable Red* negative cells to exclude non-viable cells. *P ≤ 0.05, **P ≤ 0.01, ***P ≤ 0.001, and ****P ≤ 0.0001 as determined by unpaired two-tailed *t*-test.

Several Btn and Btnl family members have been reported to regulate T cell proliferation (1–7). To investigate an analogous effect of the epithelial cell-specific Btnl molecules, we performed an *in vitro* T cell proliferation assay making use of a long-term culture system for intestinal IELs, which permits IELs to be rested as viable cells and then rapidly re-activated when stimulated via the TCR (18, 21), and the fluorescent dye CFSE, which penetrates cell membranes and couples to proteins resulting in stable, long-term intracellular retention. Using costimulation with anti-CD3 mAb, and conditions without stimulation, the effect of Btnl proteins expressed by transfected MODE-K epithelial cells was assessed on IEL responses. Although IEL proliferation was not reproducibly affected by coculture with MODE-K-Btnl in the presence of anti-CD3 activation (Figure 5A), significant increase in proliferation was observed in the absence of TCR stimulation at both 72 and 96 hours of coculture (Figures 5B,C). The proliferative effect was dependent on the presence of exogenous IL-2 or IL-15 as in the absence of these cytokines no proliferation was observed (Figure 5B). Although both Btnl1 and the Btnl1–Btnl6 heteromer were able to induce IEL proliferation, the expansion in IL-15-treated cells was considerably higher in the presence of Btnl1 (Figure 5C). The capacity to proliferate in the presence of Btnl proteins was specific for IELs as no proliferation was induced when unstimulated splenocytes were cocultured in the presence of Btnl-transfected MODE-K cells (Figure S1A in Supplementary Material). In contrast, when the coculture experiment with splenocytes was performed in the presence of anti-CD3 and anti-CD28 stimulation, the activation-induced T cell proliferation was significantly reduced in the presence of both Btnl1, confirming previously published data where Btnl1-IgG-Fc was used to evaluate Btnl1 effect on T cell activation (2), and the Btnl1–Btnl6 complex (Figure S1B in Supplementary Material).

In order to exclude that the proliferative response of IELs was due to the allogeneic conditions, we reproduced the cocultures with Btnl-expressing MODE-K cells with syngeneic H-2k IELs derived from C3H/He mice. Substantial and significant proliferation of IELs was observed in the presence of Btnl molecules in the syngeneic condition verifying that the allogeneic conditions in coculture experiments with Btnl-MODE-K cells and IELs derived from BL6 mice do not contribute to the functional outcome of IEL expansion (Figure S2 in Supplementary Material).

### MODE-K Cell Expressed Btnls Induce IFN-γ Secretion and Upregulate CD25 Expression on αβ and γδ TCR IELs

**Figure 6:**
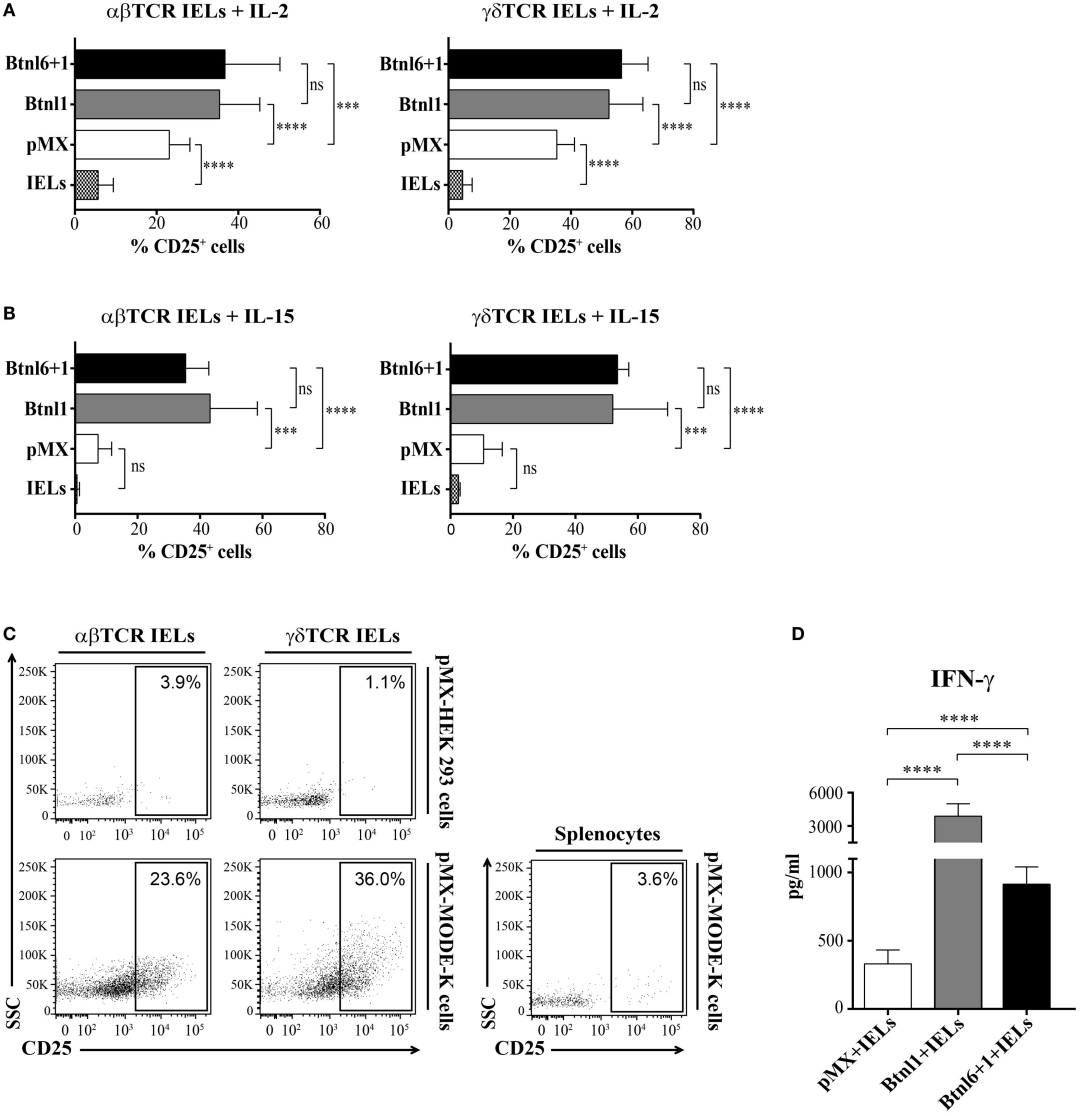
**Btnl proteins induce CD25 expression**. **(A,B)** MODE-K cells transfected with Btnl6 and Btnl1 cDNA pMX-IRES-GFP, Btnl1 cDNA pMX-IRES-GFP, or empty vector (pMX-IRES-GFP) were cocultured with IELs without anti-CD3 activation in the presence of IL-2 **(A)** or IL-15 **(B)**. CD25 expression was assessed on gated αβ TCR and γδ TCR IELs, after gating on LIVE/DEAD *Fixable Red* negative cells to exclude non-viable cells subsets and on CD45^+^ to exclude GFP^+^ MODE-K cells, after 96 h. Graphs show the mean ± SD, and data are pooled from seven independent experiments, each in duplicates, for IL-2, and two independent experiments, each performed in triplicates, for IL-15. **(C)** Culture of total IELs or splenocytes, without anti-CD3 in the presence of IL-2, with HEK 293 cells or MODE-K cells transfected with empty vector (pMX-IRES-GFP). Splenocytes and IELs gated on αβ TCR or γδ TCR IEL subsets, after gating on LIVE/DEAD *Fixable Red* negative cells to exclude non-viable cells subsets and on CD45^+^ to exclude GFP^+^ MODE-K cells, were analyzed for CD25 expression after 96 h. Plots shown are representative of seven independent experiments for IELs and MODE-K cells, and of two independent experiments for IELs and HEK 293 or splenocytes and MODE-K cells. **(D)** Culture of total IELs, without anti-CD3 in the presence of IL-15, with MODE-K cells transfected with Btnl-pMX-IRES-GFP or empty vector control (pMX-IRES-GFP). IFN-γ was assessed by cytometric bead array. Bars show the mean ± SD, and data are pooled from two independent coculture experiments, each performed in triplicates. *P ≤ 0.05, **P ≤ 0.01, ***P ≤ 0.001, and ****P ≤ 0.0001 as determined by unpaired two-tailed *t*-test.

During steady-state conditions, IELs lack or have low levels of CD25 expression (24) reflecting a resting non-proliferative state, and the cultured IELs did not express CD25 prior to coculture with the epithelial MODE-k cells (data not shown). Although MODE-K cells transfected with pMX were able to support CD25 upregulation on both CD8αβ^+^ TCRαβ^+^ IELs and CD8αα^+^ TCRγδ^+^ IELs in the presence of IL-2 (Figure 6A), the induction of CD25 was significantly enhanced when IELs were cocultured with MODE-K cells expressing Btnl1 or the Btnl1–Btnl6 heteromer (Figure 6A). In contrast, in conditions with IL-15, CD25 expression was only upregulated on IELs in the presence of the Btnl proteins and was not induced by MODE-K epithelial cells transfected with empty vector (Figure 6B). CD25 expression on IELs turned out to associate, and also to correlate, with the proliferative activity of the IELs (Figures S3A,B in Supplementary Material). The potential to induce CD25 expression was restricted to epithelial cells as IELs cocultured with HEK 293 cells in the presence of IL-2 did not become CD25^+^ (Figure 6C), and specific for IELs as splenocytes cocultured in the presence of MODE-K cells failed to upregulate CD25 (Figure 6C). Moreover, the epithelial cell-mediated upregulation of CD25 was dependent on direct cell–cell contact as CD25 induction did not occur in cocultures in which IELs and MODE-K cells were separated by transwells (Figure S3C in Supplementary Material).

We further analyzed the functional role of the Btnl proteins by investigating cytokine production in the Btnl-MODE-K cell–IEL cocultures. Cytokine profiling of supernatants by cytometrix bead array (CBA) identified significant and specific increase in the expression of IFN-γ (the induction of other IEL cytokines, e.g., TNF-α or IL-5, was largely unaffected) in cocultures with IELs and Btnl1- or Btnl1–Btnl6-transfected MODE-K epithelial cells when compared to conditions with pMX transfectants (Figure 6D). As in the case of Btnl-induced proliferation, Btnl1 was more potent in inducing IFN-γ secretion than the Btnl1–Btnl6 heteromer (Figure 6D).

### Btnl1–Btnl6 Complex Enhances the Proliferation of Vγ7Vδ4 IELs

**Figure 7:**
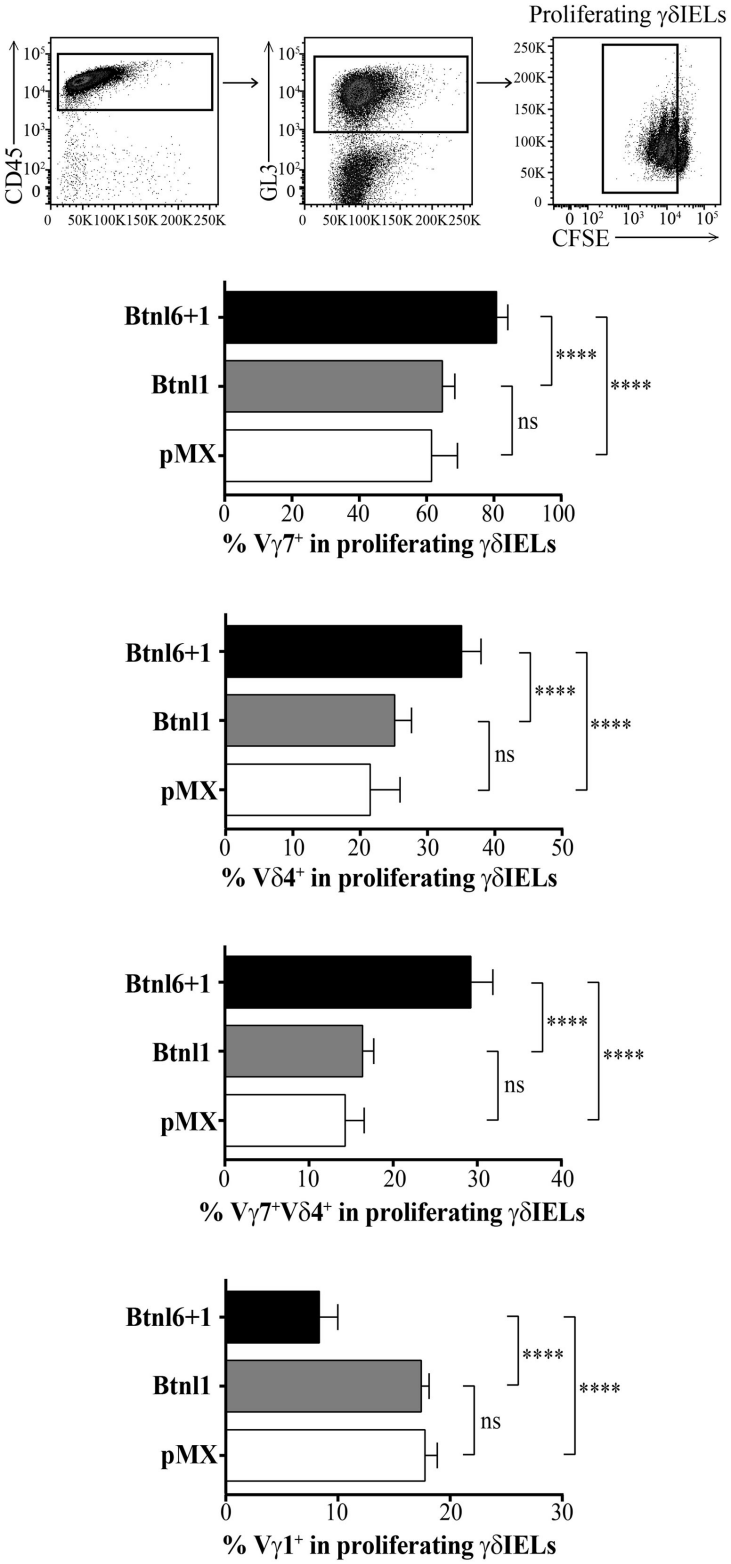
**The Btnl1–Btnl6 complex specifically induces the proliferation of IELs bearing the Vγ7Vδ4 receptor**. Murine MODE-K cells transfected with Btnl6 and Btnl1 cDNA pMX-IRES-GFP, Btnl1 cDNA pMX-IRES-GFP, or empty vector (pMX-IRES-GFP) were cocultured with CFSE-labeled IELs in the absence of anti-CD3 activation in the presence of IL-2. The proliferating population among the γδ TCR IELs in the various coculture conditions was analyzed for the expression of Vγ7, Vγ1, and Vδ4 chains. Bars show the mean ± SD, and data are pooled from four independent experiments in duplicates. *P ≤ 0.05, **P ≤ 0.01, ***P ≤ 0.001, and ****P ≤ 0.0001 as determined by unpaired two-tailed *t*-test.

In order to better characterize the functional interaction between the Btnl proteins and γδ TCR IELs, we analyzed the expression of Vγ7, Vγ1, and Vδ4, i.e., the main chains utilized in the C57BL/6 mouse strain (25), in the cocultures with Btnl-transfected MODE-K epithelial cells. While no difference in expansion of proliferating IELs expressing the Vγ7, Vγ1, and Vδ4 chains was observed in cocultures with MODE-K cells transfected with Btnl1 versus pMX, the Btnl1–Btnl6 complex specifically enhanced the proliferation of IELs bearing the Vγ7Vδ4 receptor (Figure 7). Synchronously, a significant decrease in the proportion of proliferating Vγ1-expressing IELs was observed in the cocultures, presumably as a consequence of the increase in the proportion of Vγ7^+^ IELs (Figure 7). The reverse was seen in the non-proliferating population, i.e., a higher ratio of Vγ1-expressing IELs and a lower proportion of Vγ7-bearing cells in conditions with Btnl1–Btnl6 (Figure S4 in Supplementary Material), suggesting a selective expansion of Vγ7-expressing IELs rather than, e.g., Btnl1–Btnl6 promoted cell-death of Vγ1-bearing cells.

## Discussion

Although several Btn and Btnl family members have been reported to be implicated in regulating the biology of peripheral T cells, their capacity to control tissue-associated T cells, such as IELs, is still poorly defined. The location of IELs within the epithelial compartment offers obvious opportunities for direct epithelial–T cell interaction although few molecular conduits of this have been identified in the intestinal mucosa. We have previously shown that Btnl1 and Btnl6 transcripts are highly expressed in the gut and that Btnl1 protein can be detected on the surface of small intestinal epithelial cells (18). The essentially restricted localization of Btnl1 and Btnl6 to the gut epithelium suggests that the proteins may be involved in regulating IEL-mediated immune responses, as observed for the skin epithelium resident Skint1, a close relative to Btnl1 and Btnl6 (23), that regulates epidermal γδ IEL development (26, 27), and for junctional adhesion molecule-like protein (JAML), that controls skin γδ IEL proliferation and activation (28). Indeed, we previously demonstrated that Btnl1 can regulate epithelial cell responses to activated intestinal IELs by suppressing the production of proinflammatory cytokines, such as IL-6 (18). This study further supports the function of the Btnl genes in local gut immune regulation. Using a generated anti-Btnl6 polyclonal antibody, we have characterized the expression of Btnl6 and demonstrated that the Btnl6 protein is readily expressed in the intestine where it is confined to the epithelial cells. In contrast to Btnl1 that can be detected on the cell surface of various cell lines upon transfection, and also on *ex vivo* enterocytes (18), we have shown that cell surface expression of Btnl6 on MODE-K small intestinal epithelial cells is dependent on the presence of Btnl1. Mass spectrometry of anti-Btnl1 immunoprecipitated lysates from primary small intestinal epithelial cells revealed a previously unidentified intrafamily high-molecular mass complex containing Btnl1 and Btnl6 and provided evidence that Btnl1–Btnl6 heteromerization occurs *in vivo*. Btnl1–Btnl6 heteromerization in epithelial cells may explain the inability of the generated anti-Btnl6 antibody to recognize the non-reduced form of the Btnl6 protein. Despite several attempts to generate an anti-Btnl6 antibody recognizing the native form of the Btnl6 protein, the antibodies only recognized the proteins in its reduced form, making the study of protein localization or expression on primary cells difficult. In addition to the high-molecular mass Btnl1–Btnl6 complex, the pull-down assay detected a ~130 kDa band which proved to contain Btnl1, but not other Btnl-family members, suggesting that the complex most likely includes Btnl1 homodimers. Thus, our data suggest that the proteins rather than forming heterodimers, multimerize into high-molecular weight complexes. The presence of multiple Btnl forms in the intestinal epithelium suggests that the Btnl proteins may have different functions determined by their form. Indeed, although Btnl1 alone caused IELs to divide and the cotransfection with Btnl6 did not further increase the proliferation of IELs, suggesting that the complex formation is not critical for inducing IEL division, the Btnl1–Btnl6 complex selectively regulates the expansion of Vγ7Vδ4 IELs. Furthermore, even though both Btnl1 and Btnl1–Btnl6 were able to induce proliferation and IFN-γ production by IELs, the expansion of IL-15-treated IELs and the amount of released IFN-γ was significantly higher in the presence of Btnl1. This may reflect the proteins’ different efficiency in regulating IEL function, or even indicate a negative feedback provided by Btnl6 counteracting the effect of Btnl1.

Several studies have demonstrated that Btn and Btnl proteins have the capacity to either suppress (1–6) or enhance (3, 7) costimulation-induced T cell activation and proliferation. Although we were able to confirm the previously reported suppressive effect of Btnl1 on CD3-activated peripheral T cells (2), we found no reproducible significant suppression or activation of IEL proliferation by either Btnl1 or Btnl1–Btnl6 with anti-CD3 stimulation. Instead, our results demonstrate that the epithelium-associated Btnl molecules are capable of inducing activation and proliferation of intestinal IELs in the absence of exogenous activation. The proliferative effect was dependent on the presence of IL-2 or IL-15. Although IL-2 may be available in the small intestine under steady-state conditions (29), its level is normally low in the absence of activation. In contrast, IL-15, reportedly able to enhance survival and proliferation of CD8αα^+^TCRαβ^+^ and CD8αα^+^TCRγδ^+^ IELs (30, 31), is constitutively expressed by small intestinal epithelial cells (32). Consequently, cocultures with the epithelial cell-derived IL-15 may better represent homeostatic conditions in the gut. Although IL-15 reportedly exerts functional effects on lymphocytes similar to those of IL-2, only IL-2 was able to promote the MODE-K cell-mediated upregulation of CD25 on cultured IELs. Instead, the IL-15-mediated upregulation of CD25 expression on IELs was dependent on the presence of the Btnl proteins, suggesting a synergy between IL-15 and Btnl for regulation of IEL biology.

Given that mucosal IELs are specialized T cells with a unique cellular composition and development compared to other T cells in the body, it is not unlikely that their interaction with Btnl proteins in the intraepithelial compartment would elicit responses different from those obtained in systemic T cells. IELs constitute a large T cell pool, with 1 IEL for every 5–10 epithelial cell, but little is known of the mechanisms contributing to their maintenance and turnover in the intraepithelial compartment, or of their activation requirements. A study by Stankovic et al. (33) suggested that intestinal IELs can proliferate in the absence of overt activation and thus that IEL expansion is homeostatically driven. Nevertheless, the mechanism behind the apparent TCR ligation-independent proliferation has to our knowledge not yet been fully defined. Our data implies that the homeostatic expansion of IELs, crucial for the maintenance of the local immune system, may be driven by the epithelium-specific Btnl proteins.

The Btnl proteins not only upregulate CD25 expression and promote IEL proliferation but can also enhance IFN-γ secretion by IELs. In view of our previous data, showing that Btnl1 has no effect on IEL-mediated IFN-γ production in the presence of exogenous activation (18), the finding that the Btnl proteins can enhance IFN-γ secretion in the absence of overt activation implies that the interaction between IELs and Btnl proteins may lead to different outcomes depending on local conditions, e.g., during intestinal homeostasis or inflammatory stress. Synthesis of IFN-γ under steady-state conditions *in vivo* may have a protective role in the removal of diseased or transformed epithelial cells or additionally be involved in oral tolerance (34, 35). It has additionally been demonstrated that IFN-γ secretion may act to increase the abundance of CD8^+^ IELs (36) and, thus, the Btnl-induced production of IFN-γ may contribute to the maintenance of IELs in the intestinal mucosa.

In summary, the characterization of novel determinants controlling the function of intestinal IELs provides new insights into regulation of tissue-localized T cells. The specific expression of Btnl1 and Btnl6 in intestinal epithelial cells, together with their ability to control IEL expansion and activation strongly implicates Btnl proteins in local immune responses and IEL–epithelial cell interaction pathways.

## Author Contributions

AB-F supervised the project, designed experiments and interpreted the data, and wrote the paper; CL-F designed and performed experiments, analyzed data, prepared figures and supplementary information for the manuscript, and contributed to the writing of the manuscript; JB and TP contributed equally to this work: both designed and performed experiments, analyzed data, and prepared figures for the manuscript.

## Conflict of Interest Statement

The authors declare that the research was conducted in the absence of any commercial or financial relationships that could be construed as a potential conflict of interest.

## Abbreviations

Btn, butyrophilin; Btnl, butyrophilin-like; HEK, human embryonic kidney; IEL, intraepithelial lymphocyte.
