# Supplementary material for: Murine Butyrophilin-Like 1 and Btnl6 Form Heteromeric Complexes in Small Intestinal Epithelial Cells and Promote Proliferation of Local T Lymphocytes
Source: Front Immunol. 2016 Jan 19;7:1. doi: 10.3389/fimmu.2016.00001 (PMC4717187; doi:10.3389/fimmu.2016.00001)
Supplement: Supplementary file 1 [file presentation_1.pdf]

Supplementary Material

Murine Butyrophilin-Like (Btnl) 1 and Btnl6 Form Heteromeric Complexes in Small Intestinal Epithelial Cells and Promote Proliferation of Local T Lymphocytes

Cristina Lebrero-Fernández[1], Joakim H. Bergström[2†], Thaher Pelaseyed[2†], Anna Bas-Forsberg[1*]

[1]Department of Microbiology and Immunology, Institute of Biomedicine, University of Gothenburg, Gothenburg, Sweden

[2]Department of Medical Biochemistry and Cell Biology, Institute of Biomedicine, University of Gothenburg, Gothenburg, Sweden

[†]Contributed equally to this study

[*]**Correspondence**: Dr. Anna Bas-Forsberg, Department of Microbiology and Immunology, Institute of Biomedicine, University of Gothenburg, Medicinaregatan 7A, 413 90 Gothenburg, Sweden; anna.forsberg1@gu.se

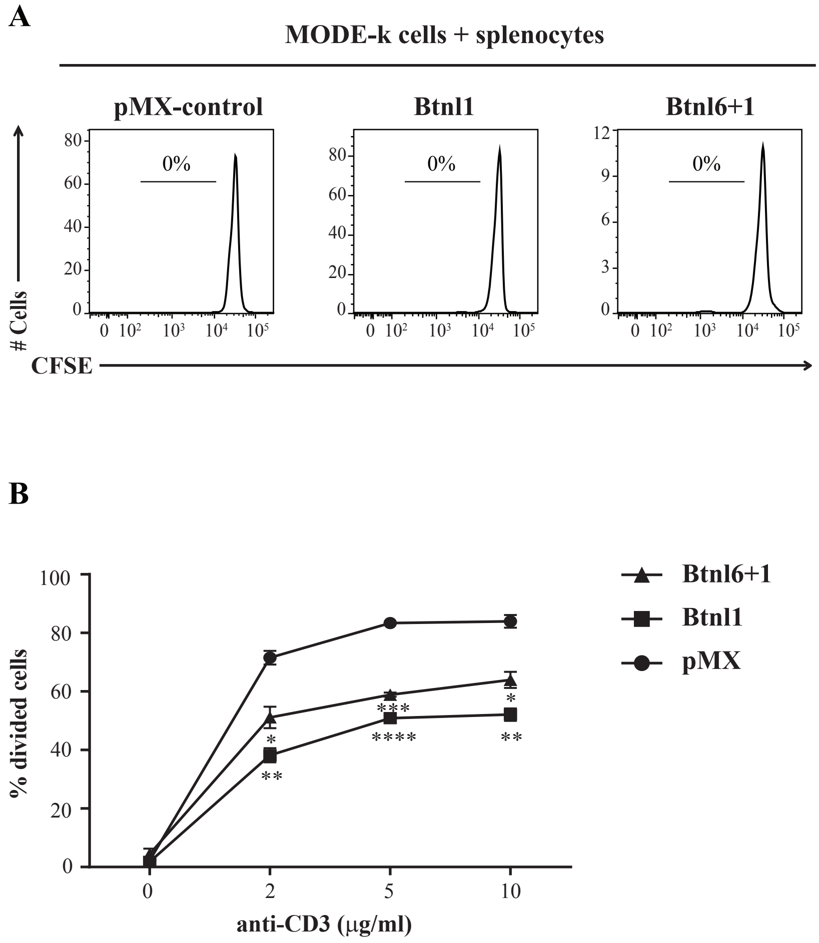

Figure S1. (A) Btnl proteins do not induce proliferation of unactivated splenocytes. MODE-K cells transfected with Btnl6 and Btnl1 cDNA pMX-IRES-GFP, Btnl1 cDNA pMX-IRES-GFP, or empty vector (pMX-IRES-GFP) were cocultured with CFSE-labeled splenocytes in the presence of IL-2. Splenocytes were left to proliferate and cell division was monitored after 96 hours. Histograms shown are representative of two independent experiments, each performed in duplicates. **(B) Btnl1 inhibits proliferation of activated peripheral T cells.** MODE-K cells transfected with Btnl6 and Btnl1 cDNA pMX-IRES-GFP, Btnl1 cDNA pMX-IRES-GFP, or empty vector (pMX-IRES-GFP) were cocultured with CFSE-labeled peripheral T cells in the presence of anti-CD3 and anti-CD28 (2 μg/ml) activation. Peripheral T cells were left to proliferate and cell division was monitored after 72 hours. Data are pooled from two independent experiments. Cells were gated on LIVE/DEAD *Fixable Red* negative cells to exclude non-viable cells and on CD45+ to exclude GFP+ MODE-K cells. *P≤0.05, **P≤0.01, ***P≤0.001 and ****P≤0.0001 as determined by unpaired two-tailed t-test.

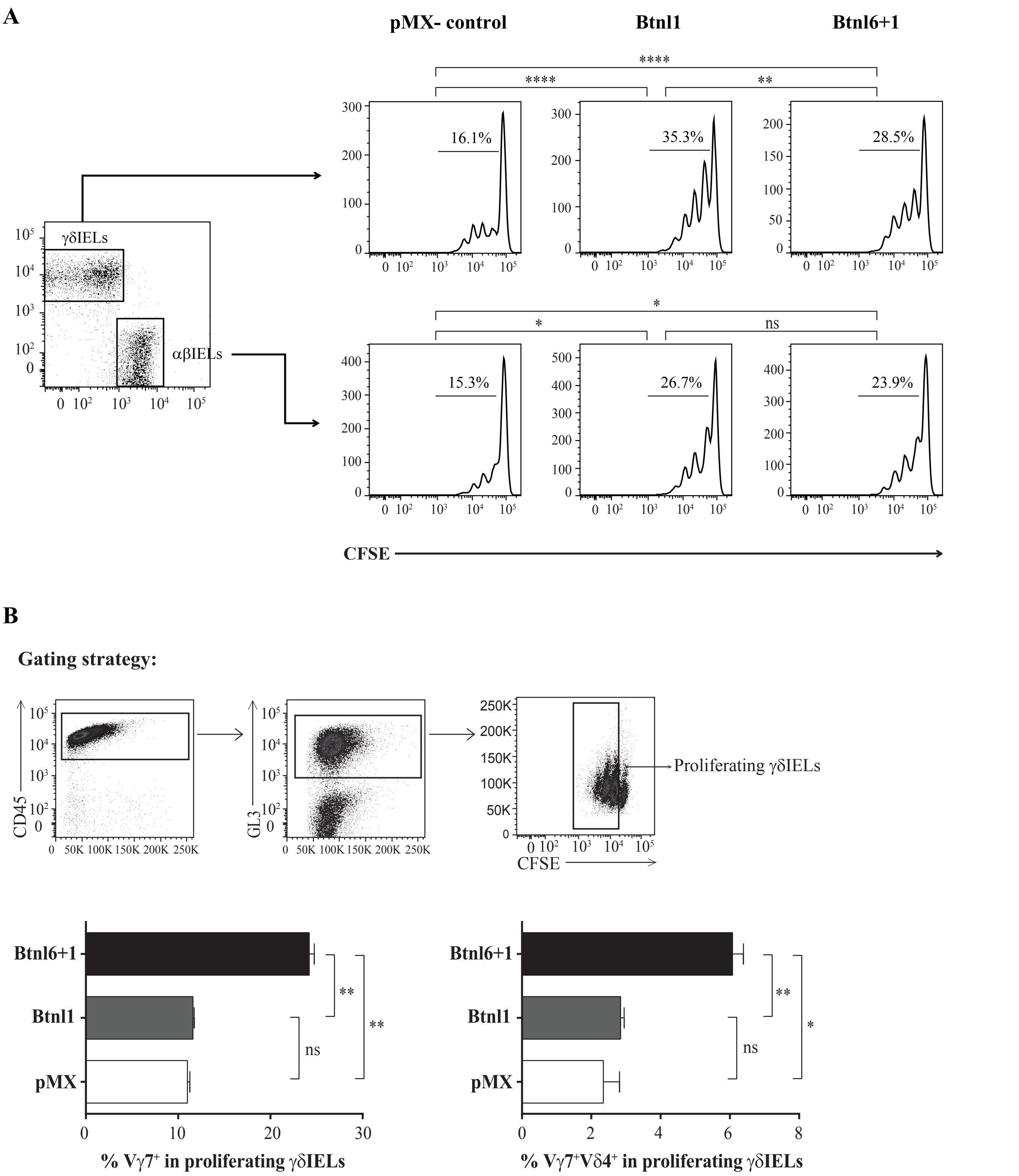

Figure S2. IEL proliferation in cocultures with Btnl-MODE-K cells and syngeneic H-2k IELs. MODE-K cells transfected with Btnl6 and Btnl1 cDNA pMX-IRES-GFP, Btnl1 cDNA pMX-IRES-GFP, or empty vector (pMX-IRES-GFP) were cocultured with CFSE-labeled C3H/HeN derived IELs in the absence of anti-CD3 activation in the presence of IL-2. **(A)** IELs were left to proliferate for 96 hours and cell division was monitored in αβTCR and γδTCR IEL subsets. CFSE-stained IEL proliferation is shown as the percentage of divided cells. Histograms shown are representative of two independent experiments, each performed in duplicates. **(B)** The proliferating population among the γδTCR IELs in the various coculture conditions was analyzed for the expression of Vγ7 and Vδ4 chains. Bars show the mean ± SD and data are pooled from two independent experiments in duplicates. Cells were gated on LIVE/DEAD *Fixable Red* negative cells to exclude non-viable cells and on CD45+ to exclude GFP+ MODE-K cells.*P≤0.05, **P≤0.01, ***P≤0.001 and ****P≤0.0001 as determined by unpaired two-tailed t-test.

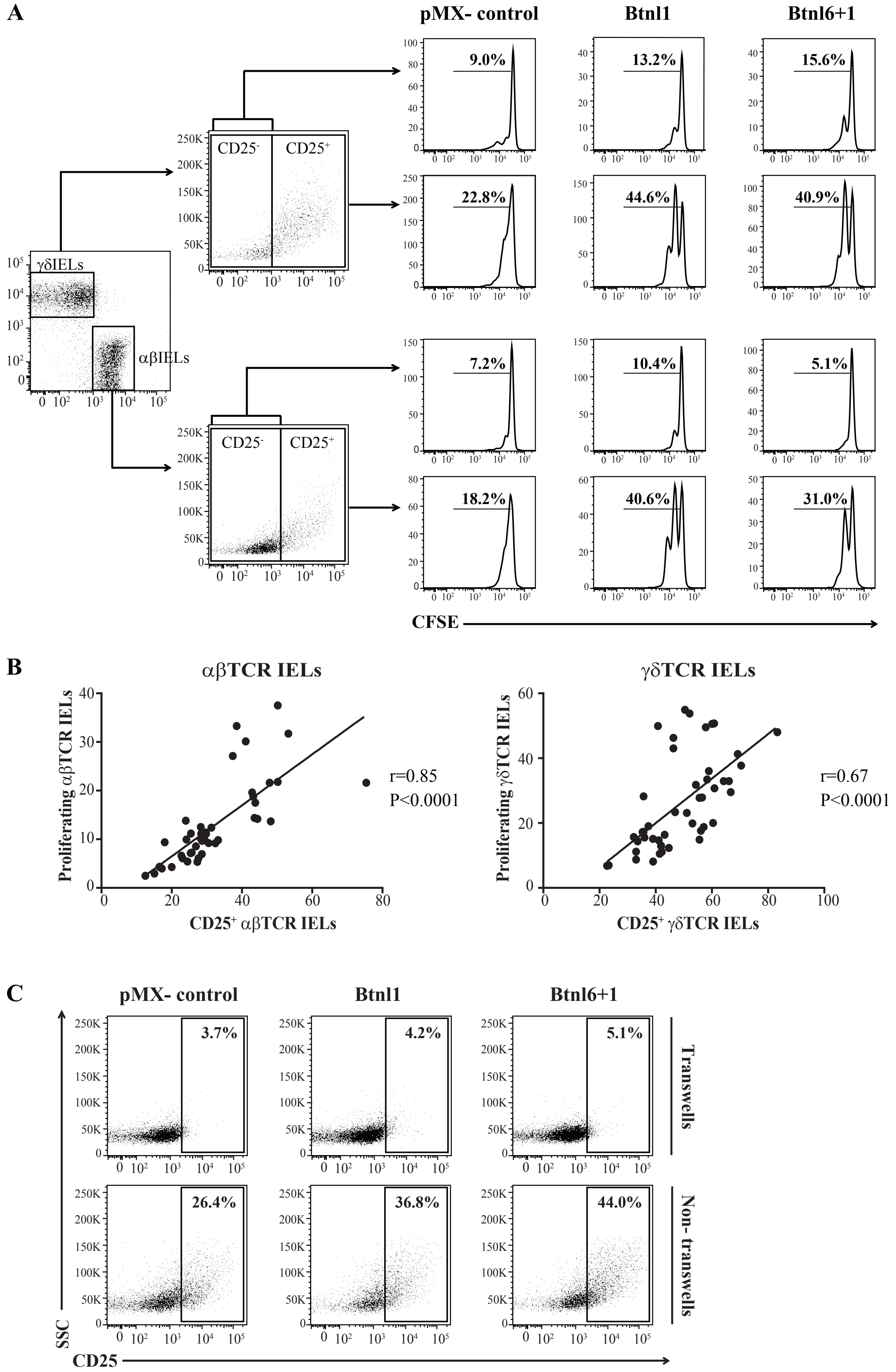

Figure S3. (A) Btnl1-Btnl6 and Btnl1 augment proliferation of CD25+ IELs. MODE-K cells transfected with Btnl6 and Btnl1cDNA pMX-IRES-GFP, Btnl1 cDNA pMX-IRES-GFP, or empty vector (pMX-IRES-GFP) were cocultured with CFSE-labeled IELs in the absence of anti-CD3 activation in the presence of IL-2. IELs were left to proliferate for 96 hours and cell division was monitored in αβTCR and γδ TCR IEL subsets. CFSE-stained IEL proliferation is shown as the percentage of divided cells. Histograms shown are representative of seven independent experiments, each performed in duplicates. **(B) Cellular proliferation correlates with the expression of CD25.** Levels of CD25 expressed on αβTCR and γδTCR IEL subsets were plotted against the proliferation at 96 hour time point. Data are from 7 independent experiments, each performed in duplicates. Correlation between CD25 expression and IEL proliferation was determined using the Spearman correlation test. **(C) Epithelial-cell mediated activation of IELs is dependent on direct cell-cell contact.** Total IELs were cocultured, without anti-CD3 in the presence of IL-2, with MODE-K cells transfected with Btnl-IRES-GFP or pMX-IRES-GFP. IELs and MODE-K epithelial cells were cultured with or without separation by transwells. IELs were analyzed for CD25 expression after 96 hours. Plots shown are representative of two independent experiments, each performed in duplicates. Cells were gated on LIVE/DEAD *Fixable Red* negative cells to exclude non-viable cells and on CD45+ to exclude GFP+ MODE-K cells.

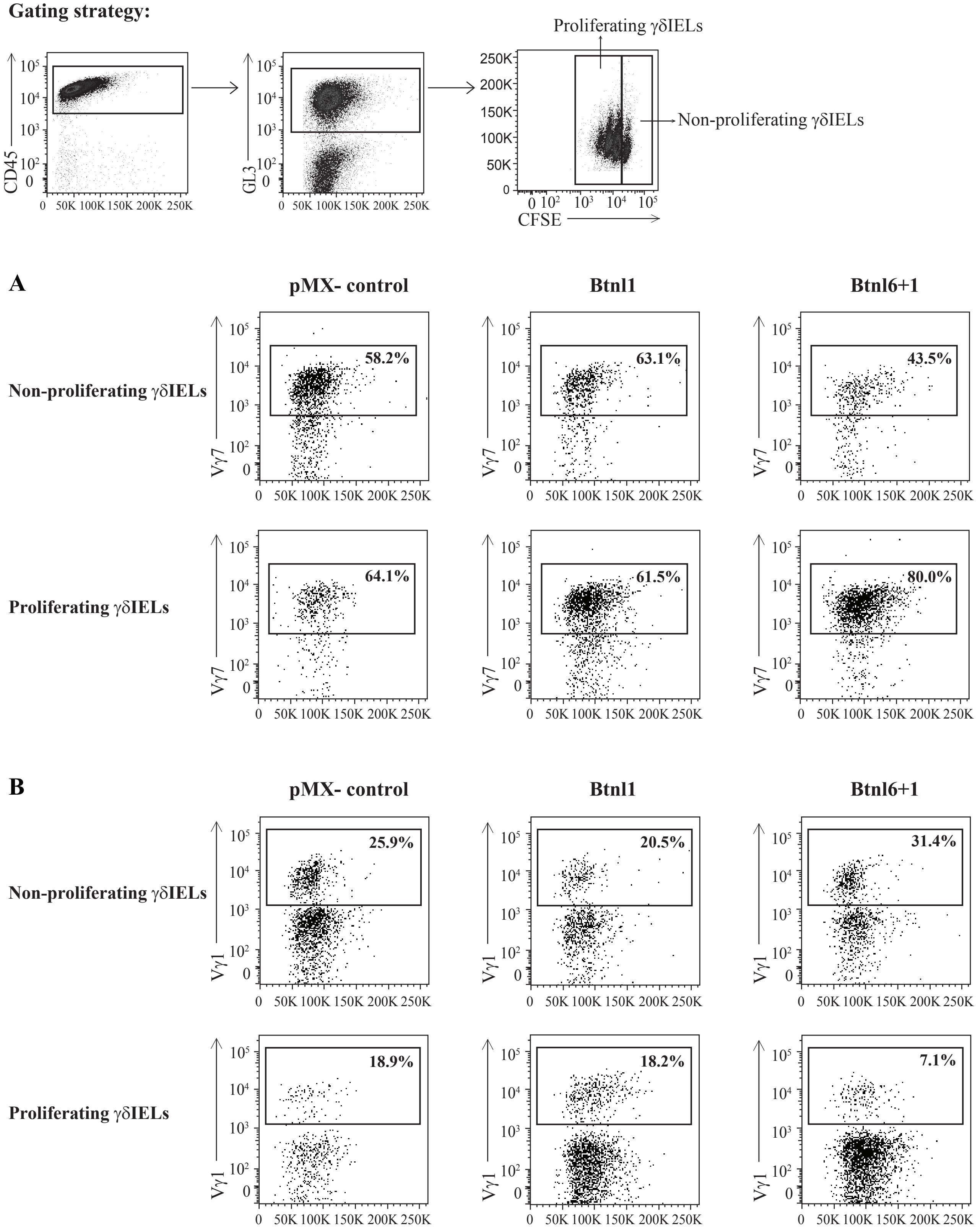

Figure S4. Representation of Vγ7- and Vγ1 expressing IELs among non-proliferating and proliferating γδ TCR IELs. Murine MODE-K cells transfected with Btnl6 and Btnl1 cDNA pMX-IRES-GFP, Btnl1 cDNA pMX-IRES-GFP, or empty vector (pMX-IRES-GFP) were cocultured with CFSE-labeled IELs in the absence of anti-CD3 activation in the presence of IL-2. The non-proliferating and proliferating populations among the γδ TCR IELs in the various coculture conditions were analyzed for the expression of Vγ7 **(A)** and Vγ1 chains **(B)**. Plots shown are representative of four independent experiments, each perfomed in duplicates.

Table S1. Complete list of proteins identified by mass spectrometry.

Band 1					
**AC number**	**Protein name**	**Protein score**	**Protein mass (D)**	**Queries matched**	**Unique peptides**
gi|16303309	type II keratin 5 [Mus musculus]	1158	61743	73	20
gi|293686	epidermal keratin subunit II [Mus musculus]	790	59414	46	4
gi|51092293	keratin, type II cytoskeletal 1b [Mus musculus]	770	61322	35	2
gi|387397	epidermal keratin subunit I, partial [Mus musculus]	679	57807	25	6
gi|54607171	keratin, type II cytoskeletal 6A [Mus musculus]	491	59299	35	3
gi|46485130	TPA_exp: keratin Kb40 [Mus musculus]	485	85186	14	3
gi|21489935	keratin, type I cytoskeletal 14 [Mus musculus]	455	52834	31	2
gi|6680604	keratin, type I cytoskeletal 16 [Mus musculus]	449	51574	15	3
gi|398168	keratin 2 epidermis [Mus musculus]	448	70934	25	7
gi|29789317	keratin, type II cytoskeletal 75 [Mus musculus]	422	59704	24	4
gi|7106335	keratin, type I cytoskeletal 17 [Mus musculus]	402	48132	26	2
gi|38503465	keratin 17n [Mus musculus]	375	50146	30	7
gi|470674	collagen pro-alpha-1 type I chain [Mus musculus]	258	137859	22	12
gi|13542680	Tubulin, beta 2C [Mus musculus]	257	49783	27	3
gi|16307541	Atp1a1 protein, partial [Mus musculus]	254	108338	21	12
gi|148672085	mCG144996, partial [Mus musculus]	246	38119	20	3
gi|28395018	junction plakoglobin [Mus musculus]	243	81749	20	17
gi|6680748	ATP synthase subunit alpha, mitochondrial precursor [Mus musculus]	236	59716	15	12
gi|47059013	keratin, type II cytoskeletal 73 [Mus musculus]	234	58875	14	4
gi|6678469	tubulin alpha-1C chain [Mus musculus]	225	49877	16	10
gi|270309140	nucleoprotein TPR [Mus musculus]	211	273824	17	15
gi|157823889	actin, gamma-enteric smooth muscle [Mus musculus]	195	41850	13	2
gi|189409106	butyrophilin-like protein 1 precursor [Mus musculus]	193	57689	10	8
gi|21703836	putative hexokinase HKDC1 [Mus musculus]	192	102193	15	4
gi|22094075	ADP/ATP translocase 2 [Mus musculus]	182	32910	17	6
gi|17978023	nonmuscle heavy chain myosin II-A [Mus musculus]	161	226217	20	14
gi|15215161	Microsomal triglyceride transfer protein [Mus musculus]	155	99051	14	14
gi|148690794	myosin, heavy polypeptide 14, isoform CRA_b, partial [Mus musculus]	150	227849	21	2
gi|58218988	long-chain-fatty-acid--CoA ligase 5 [Mus musculus]	145	76157	13	12
gi|556301	elongation factor Tu [Mus musculus]	134	50132	15	12
gi|122728	RecName: Full=Hemoglobin subunit epsilon-Y2 [Mus musculus]	133	16126	4	2
gi|37360088	mKIAA0778 protein [Mus musculus]	110	112220	11	3
gi|21312260	aldehyde dehydrogenase X, mitochondrial precursor [Mus musculus]	109	57516	7	6
gi|74144331	unnamed protein product [Mus musculus]	101	116797	10	2
gi|45597453	long-chain fatty acid transport protein 4 [Mus musculus]	100	72272	8	8
gi|74192292	unnamed protein product [Mus musculus]	91	139170	5	5
gi|19526818	phosphate carrier protein, mitochondrial precursor [Mus musculus]	84	39606	2	2
gi|160420143	butyrophilin-like 6 precursor [Mus musculus]	84	60980	4	3
gi|568935994	PREDICTED: protein RUFY3 isoform X4 [Mus musculus]	82	66907	9	7
gi|2961456	RNA helicase A [Mus musculus]	79	149489	7	6
gi|21703962	peroxisome assembly factor 2 [Mus musculus]	75	104483	5	4
gi|1167510	TI-225 [Mus musculus]	73	14167	6	2
gi|18252782	antithrombin-III precursor [Mus musculus]	71	51971	7	6

gi|3329496	heterogenous nuclear ribonucleoprotein U [Mus musculus]	70	87837	3	3
gi|33859811	trifunctional enzyme subunit alpha, mitochondrial precursor [Mus musculus]	69	82617	8	7
gi|163838641	ATP synthase subunit gamma, mitochondrial isoform a [Mus musculus]	68	32865	3	3
gi|6677809	40S ribosomal protein S6 [Mus musculus]	68	28663	2	2
gi|124248512	carbamoyl-phosphate synthase [ammonia], mitochondrial precursor [Mus musculus]	66	164514	7	7
gi|387114	complement component C3 prepropeptide, last, partial [Mus musculus]	64	186365	9	8
gi|73921246	RecName: Full=Niemann-Pick C1-like protein 1 [Mus musculus]	64	147037	2	2
gi|26340966	unnamed protein product [Mus musculus]	64	68678	3	3
gi|148670639	mCG20503, isoform CRA_a [Mus musculus]	61	40134	4	2
gi|55153885	Glyceraldehyde-3-phosphate dehydrogenase [Mus musculus]	60	35751	7	3
gi|32129201	desmoglein-1-beta precursor [Mus musculus]	59	114382	9	6
gi|27370092	elongation factor Tu, mitochondrial isoform 1 [Mus musculus]	59	49477	6	6
gi|6996913	annexin A2 [Mus musculus]	59	38652	3	3
gi|74195497	unnamed protein product [Mus musculus]	58	52201	2	2
gi|60360584	mKIAA4195 protein [Mus musculus]	57	116525	6	6
gi|33990640	Acaca protein, partial [Mus musculus]	51	95907	4	4
gi|26344812	unnamed protein product [Mus musculus]	51	66728	3	3
gi|54130	sodium/potassium ATPase beta subunit [Mus musculus]	49	31462	3	3
gi|34538601	cytochrome c oxidase subunit II (mitochondrion) [Mus musculus]	46	25959	3	3
gi|169790797	IgGFc-binding protein precursor [Mus musculus]	46	275058	7	4
gi|148694672	mCG134629, isoform CRA_a [Mus musculus]	46	25956	4	2
gi|26389323	unnamed protein product [Mus musculus]	46	98236	10	8
gi|6681115	cytochrome P450 3A13 [Mus musculus]	45	57455	4	4
gi|26346504	unnamed protein product [Mus musculus]	45	17719	2	2
gi|13430890	histone H1.4 [Mus musculus]	44	21964	3	2
gi|26344109	unnamed protein product [Mus musculus]	44	22132	4	2
gi|6671664	calnexin precursor [Mus musculus]	43	67236	6	6
gi|124486712	ribosome-binding protein 1 isoform a [Mus musculus]	43	158301	5	3
gi|124487037	unconventional myosin-Ia [Mus musculus]	43	118620	8	8
gi|9790055	mitochondrial carrier homolog 2 [Mus musculus]	42	33477	4	4
gi|398050	ribosomal protein L18 [Mus musculus]	41	21585	5	4
gi|12846949	unnamed protein product [Mus musculus]	40	47108	5	6
gi|6755372	40S ribosomal protein S3 [Mus musculus]	39	26657	2	2
gi|6754976	peroxiredoxin-1 [Mus musculus]	39	22162	3	3
gi|74212085	unnamed protein product [Mus musculus]	39	20620	2	2
gi|1527176	ribosomal protein S26 [Mus musculus]	39	12984	2	2
gi|21704096	TAR DNA-binding protein 43 isoform 1 [Mus musculus]	38	44519	2	2
gi|124249090	keratin, type II cytoskeletal 80 [Mus musculus]	36	50629	7	4
gi|29145087	Ogdh protein [Mus musculus]	35	116043	2	2
gi|21313308	heterogeneous nuclear ribonucleoprotein M isoform a [Mus musculus]	35	77597	9	9
gi|148678066	mCG4624, isoform CRA_a [Mus musculus]	35	95811	2	2
gi|31127122	Lymphoid-restricted membrane protein [Mus musculus]	34	59404	5	2
gi|74219810	unnamed protein product [Mus musculus]	34	94665	11	2
gi|568941699	PREDICTED: maltase-glucoamylase, intestinal isoform X2 [Mus musculus]	34	410151	6	5
gi|6755965	voltage-dependent anion-selective channel protein 2 [Mus musculus]	33	31713	3	3

gi|6679731	coagulation factor V precursor [Mus musculus]	33	247076	2	2
gi|148673027	sphingomyelin phosphodiesterase 2, neutral, isoform CRA_a [Mus musculus]	33	26296	2	2
gi|9790073	cadherin-17 precursor [Mus musculus]	32	91588	6	6
gi|558532	alpha-2,3-sialyltransferase [Mus musculus]	32	38020	3	3
gi|1864007	tyrosine kinase [Mus musculus]	32	174808	3	3
gi|700275391	anti-lox-1 15C4 heavy chain [Mus musculus]	30	51300	5	2
gi|16359267	Scavenger receptor class A, member 5 (putative) [Mus musculus]	30	53603	4	2
gi|90508	gelsolin, cytosolic [Mus musculus]	30	80827	2	2
gi|6679939	glyceraldehyde-3-phosphate dehydrogenase, testis-specific isoform 2 [Mus musculus]	29	47412	3	2
gi|7576745	fanconi anemia complementation group A [Mus musculus]	28	12355	3	2
gi|6754524	L-lactate dehydrogenase A chain isoform 1 [Mus musculus]	27	36475	3	3
gi|957204	meprin beta-subunit [Mus musculus]	27	79498	2	2
gi|5738222	flavo-binding protein [Mus musculus]	24	50274	5	5
gi|12836310	unnamed protein product [Mus musculus]	23	38974	2	2
gi|6679587	ras-related protein Rab-1A [Mus musculus]	23	22663	2	2
gi|148709948	mCG18776 [Mus musculus]	22	89298	3	2
gi|20071589	Bdh1 protein, partial [Mus musculus]	21	28697	4	3
gi|148690354	mCG146227, partial [Mus musculus]	21	10140	2	2
gi|6680720	ADP-ribosylation factor 4 [Mus musculus]	20	20384	2	2
gi|26348913	unnamed protein product [Mus musculus]	20	54391	3	3
gi|148676955	mCG118787 [Mus musculus]	19	24412	2	2
gi|12835990	unnamed protein product [Mus musculus]	17	75427	2	2
gi|568969228	PREDICTED: uncharacterized protein LOC102635990 isoform X2 [Mus musculus]	17	551505	8	7
gi|2329849	rabaptin-5 [Mus musculus]	16	99490	3	3
gi|148683908	mCG3370 [Mus musculus]	16	16490	2	2

**Band 2**					
gi|16303309	type II keratin 5 [Mus musculus]	1025	61743	68	18
gi|387397	epidermal keratin subunit I, partial [Mus musculus]	932	57807	41	6
gi|51092293	keratin, type II cytoskeletal 1b [Mus musculus]	720	61322	36	4
gi|293686	epidermal keratin subunit II [Mus musculus]	655	59414	39	4
gi|148707490	DEAH (Asp-Glu-Ala-His) box polypeptide 9, isoform CRA_b, partial [Mus musculus]	588	149638	33	28
gi|22164776	keratin, type II cytoskeletal 79 [Mus musculus]	567	57517	30	2
gi|309215	EndoA~ cytokeratin (5~ end put.) [Mus musculus]	538	53210	31	2
gi|145966692	keratin, type I cuticular Ha1 [Mus musculus]	464	47087	23	4
gi|46485130	TPA_exp: keratin Kb40 [Mus musculus]	458	85186	17	4
gi|111308159	Keratin 2 [Mus musculus]	457	70880	25	2
gi|21489935	keratin, type I cytoskeletal 14 [Mus musculus]	443	52834	25	4
gi|29789317	keratin, type II cytoskeletal 75 [Mus musculus]	365	59704	21	2
gi|238231425	keratin, type I cuticular Ha5 [Mus musculus]	331	50497	18	6
gi|7106335	keratin, type I cytoskeletal 17 [Mus musculus]	320	48132	24	3
gi|38503465	keratin 17n [Mus musculus]	307	50146	22	6
gi|28316760	histone H2B type 1-B [Mus musculus]	295	13944	11	2
gi|12844116	unnamed protein product [Mus musculus]	292	46064	17	2
gi|4103156	hair keratin basic 5 [Mus musculus]	291	55736	24	6
gi|13386452	histone H2B type 3-A [Mus musculus]	287	13986	10	2
gi|190194418	desmoplakin [Mus musculus]	271	332706	39	36

gi|148672085	mCG144996, partial [Mus musculus]	262	38119	16	3
gi|47059013	keratin, type II cytoskeletal 73 [Mus musculus]	223	58875	15	3
gi|6680606	keratin, type I cytoskeletal 19 [Mus musculus]	217	44515	12	2
gi|809561	gamma-actin [Mus musculus]	203	40992	14	2
gi|133777677	Hist2h4 protein, partial [Mus musculus]	196	11303	9	7
gi|28395018	junction plakoglobin [Mus musculus]	193	81749	19	16
gi|2598562	BiP [Mus musculus]	167	72433	10	8
gi|6996913	annexin A2 [Mus musculus]	130	38652	5	5
gi|16307541	Atp1a1 protein, partial [Mus musculus]	109	108338	8	4
gi|148677501	ATP synthase, H+ transporting, mitochondrial F1 complex, alpha subunit, isoform 1, isoform CRA_e [Mus musculus]	105	54561	12	10
gi|57013102	RecName: Full=Protein-glutamine gamma-glutamyltransferase K [Mus musculus]	101	89769	7	7
gi|309319	heat shock protein 70 cognate [Mus musculus]	100	70793	5	2
gi|13488601	type II hair keratin [Mus musculus]	96	57076	11	5
gi|23272966	Atp5b protein, partial [Mus musculus]	95	56632	7	7
gi|17160984	RUN and FYVE domain containing 3 [Mus musculus]	87	52955	5	5
gi|22094075	ADP/ATP translocase 2 [Mus musculus]	85	32910	11	6
gi|189409106	butyrophilin-like protein 1 precursor [Mus musculus]	81	57689	4	3
gi|556301	elongation factor Tu [Mus musculus]	79	50132	7	6
gi|26344914	unnamed protein product [Mus musculus]	64	29170	6	3
gi|30425250	beta-actin-like protein 2 [Mus musculus]	63	41977	7	3
gi|26389323	unnamed protein product [Mus musculus]	61	98236	5	5
gi|1929447	microsomal triglyceride transfer protein [Mus musculus]	61	99080	5	5
gi|3065927	14-3-3 protein sigma [Mus musculus]	58	27696	8	4
gi|1167510	TI-225 [Mus musculus]	58	14167	3	2
gi|309122	preprocomplement component C3 [Mus musculus]	57	186364	3	3
gi|18252782	antithrombin-III precursor [Mus musculus]	55	51971	4	4
gi|109157563	Chain K, 2.9 Angstrom X-Ray Structure Of Hybrid Macroh2a Nucleosomes [Mus musculus]	51	16152	6	4
gi|10946666	gasdermin-A [Mus musculus]	51	49562	4	4
gi|169790797	IgGFc-binding protein precursor [Mus musculus]	51	275058	5	3
gi|12851187	unnamed protein product [Mus musculus]	50	49496	6	2
gi|124249090	keratin, type II cytoskeletal 80 [Mus musculus]	48	50629	10	6
gi|32129201	desmoglein-1-beta precursor [Mus musculus]	47	114382	5	4
gi|6678469	tubulin alpha-1C chain [Mus musculus]	46	49877	6	6
gi|196976	Ig kappa-chain V-region (VJ), partial [Mus musculus]	45	11552	2	2
gi|54130	sodium/potassium ATPase beta subunit [Mus musculus]	44	31462	2	2
gi|19527174	splicing factor 3B subunit 3 [Mus musculus]	44	135465	4	4
gi|148664532	desmoglein 4, partial [Mus musculus]	44	102750	3	3
gi|1864007	tyrosine kinase [Mus musculus]	43	174808	4	4
gi|51301	unnamed protein product [Mus musculus]	41	15396	3	2
gi|6755372	40S ribosomal protein S3 [Mus musculus]	41	26657	2	2
gi|458422	polymeric immunoglobulin receptor [Mus musculus]	39	85045	3	3
gi|74152002	unnamed protein product [Mus musculus]	38	38651	2	2
gi|74208825	unnamed protein product [Mus musculus]	38	51101	3	3
gi|387111	carbohydrate binding protein 35, partial [Mus musculus]	37	27551	3	3
gi|700275391	anti-lox-1 15C4 heavy chain [Mus musculus]	34	51300	9	3
gi|6755594	116 kDa U5 small nuclear ribonucleoprotein component isoform a [Mus musculus]	33	109291	3	3
gi|407341	mitochondrial stress-70 protein [Mus musculus]	33	73483	2	2

gi|10946932	nuclear pore complex protein Nup160 [Mus musculus]	32	158130	2	2
gi|42405896	acetyl-CoA carboxylase 1 [Mus musculus]	31	264953	4	3
gi|18079339	aconitate hydratase, mitochondrial precursor [Mus musculus]	31	85410	2	2
gi|12833183	unnamed protein product [Mus musculus]	30	28472	3	3
gi|124487037	unconventional myosin-Ia [Mus musculus]	30	118620	7	7
gi|46401561	peptidylarginine deiminase, type IV [Mus musculus]	27	74260	3	3
gi|74219278	unnamed protein product [Mus musculus]	26	58429	3	3
gi|7106325	glypican-6 isoform 2 precursor [Mus musculus]	26	63016	2	2
gi|9845257	histone H1.2 [Mus musculus]	25	21254	2	2
gi|21703836	putative hexokinase HKDC1 [Mus musculus]	24	102193	6	5
gi|58218988	long-chain-fatty-acid--CoA ligase 5 [Mus musculus]	23	76157	3	2
gi|148694291	mCG16398 [Mus musculus]	23	24163	2	2
gi|148690562	mCG50669 [Mus musculus]	22	31520	2	2
gi|74219810	unnamed protein product [Mus musculus]	22	94665	5	3
gi|53169	GTP binding protein [Mus musculus]	21	113531	4	4
gi|12845995	unnamed protein product [Mus musculus]	21	56150	8	4
gi|9790161	plakophilin-1 [Mus musculus]	20	80844	3	3
gi|40787826	Eml4 protein [Mus musculus]	19	103989	3	3
gi|148710008	mCG16928 [Mus musculus]	19	26194	2	2
gi|16716569	protease, serine, 1 precursor [Mus musculus]	19	26118	4	2
gi|62945396	ras and EF-hand domain-containing protein homolog [Mus musculus]	18	70709	2	2

**Band 3**					
gi|16303309	type II keratin 5 [Mus musculus]	1317	61743	87	22
gi|387397	epidermal keratin subunit I, partial [Mus musculus]	1178	57807	53	9
gi|21489935	keratin, type I cytoskeletal 14 [Mus musculus]	1047	52834	40	3
gi|111308159	Keratin 2 [Mus musculus]	801	70880	46	10
gi|293686	epidermal keratin subunit II [Mus musculus]	788	59414	52	4
gi|148690794	myosin, heavy polypeptide 14, isoform CRA_b, partial [Mus musculus]	769	227849	49	42
gi|154090941	keratin, type I cytoskeletal 42 [Mus musculus]	722	50102	31	7
gi|51092293	keratin, type II cytoskeletal 1b [Mus musculus]	707	61322	41	3
gi|904215	cytokeratin 15 [Mus musculus]	640	49129	40	2
gi|6680604	keratin, type I cytoskeletal 16 [Mus musculus]	612	51574	24	4
gi|22164776	keratin, type II cytoskeletal 79 [Mus musculus]	609	57517	38	2
gi|54607171	keratin, type II cytoskeletal 6A [Mus musculus]	566	59299	44	3
gi|29789317	keratin, type II cytoskeletal 75 [Mus musculus]	550	59704	38	3
gi|309215	EndoA~ cytokeratin (5~ end put.) [Mus musculus]	526	53210	36	2
gi|7106335	keratin, type I cytoskeletal 17 [Mus musculus]	465	48132	28	3
gi|31980832	keratin, type II cuticular Hb5 [Mus musculus]	428	55723	29	7
gi|47059013	keratin, type II cytoskeletal 73 [Mus musculus]	399	58875	25	3
gi|28395018	junction plakoglobin [Mus musculus]	325	81749	22	18
gi|148672085	mCG144996, partial [Mus musculus]	316	38119	25	3
gi|74180977	unnamed protein product [Mus musculus]	266	234034	30	19
gi|145966692	keratin, type I cuticular Ha1 [Mus musculus]	250	47087	16	2
gi|6996913	annexin A2 [Mus musculus]	213	38652	10	9
gi|32129201	desmoglein-1-beta precursor [Mus musculus]	212	114382	9	4
gi|46485018	TPA_exp: type-II keratin Kb25 [Mus musculus]	211	54622	24	2
gi|4103158	hair keratin acidic 5 [Mus musculus]	185	47729	11	2

gi|291575137	keratin, type I cuticular Ha6 [Mus musculus]	182	52757	10	3
gi|47523977	keratin, type II cytoskeletal 72 [Mus musculus]	179	56715	16	2
gi|46485130	TPA_exp: keratin Kb40 [Mus musculus]	176	85186	8	3
gi|133777677	Hist2h4 protein, partial [Mus musculus]	155	11303	8	5
gi|74139457	unnamed protein product [Mus musculus]	154	59656	9	7
gi|6678469	tubulin alpha-1C chain [Mus musculus]	148	49877	10	4
gi|16307541	Atp1a1 protein, partial [Mus musculus]	133	108338	8	5
gi|568991513	PREDICTED: keratin, type II cuticular Hb4 isoform X1 [Mus musculus]	126	60517	13	2
gi|26340966	unnamed protein product [Mus musculus]	122	68678	6	5
gi|2623222	ATP synthase beta-subunit [Mus musculus]	112	56344	4	2
gi|31745725	small proline rich protein-like 1 protein [Mus musculus]	100	9752	6	2
gi|6754152	histidine ammonia-lyase [Mus musculus]	95	72212	3	3
gi|21312260	aldehyde dehydrogenase X, mitochondrial precursor [Mus musculus]	95	57516	10	9
gi|22094075	ADP/ATP translocase 2 [Mus musculus]	89	32910	10	5
gi|556301	elongation factor Tu [Mus musculus]	86	50132	9	8
gi|5738222	flavo-binding protein [Mus musculus]	76	50274	3	3
gi|13488601	type II hair keratin [Mus musculus]	73	57076	7	3
gi|57013102	RecName: Full=Protein-glutamine gamma-glutamyltransferase K [Mus musculus]	72	89769	7	7
gi|27370092	elongation factor Tu, mitochondrial isoform 1 [Mus musculus]	71	49477	2	2
gi|84370015	filaggrin-2 [Mus musculus]	70	251246	4	2
gi|148676670	heat shock 70kD protein 5 (glucose-regulated protein), isoform CRA_b [Mus musculus]	69	56257	5	2
gi|3986752	Hsc70t [Mus musculus]	69	60982	3	2
gi|71892420	olfactomedin-4 precursor [Mus musculus]	64	60929	5	5
gi|9790161	plakophilin-1 [Mus musculus]	61	80844	2	2
gi|124249090	keratin, type II cytoskeletal 80 [Mus musculus]	59	50629	8	5
gi|1167510	TI-225 [Mus musculus]	45	14167	2	2
gi|26376612	unnamed protein product [Mus musculus]	41	37076	4	3
gi|148678066	mCG4624, isoform CRA_a [Mus musculus]	40	95811	4	2
gi|7948999	peroxiredoxin-4 precursor [Mus musculus]	39	31033	2	2
gi|6754976	peroxiredoxin-1 [Mus musculus]	39	22162	2	2
gi|26344812	unnamed protein product [Mus musculus]	37	66728	4	3
gi|58218988	long-chain-fatty-acid--CoA ligase 5 [Mus musculus]	37	76157	2	2
gi|74206882	unnamed protein product [Mus musculus]	36	22068	4	3
gi|1929447	microsomal triglyceride transfer protein [Mus musculus]	34	99080	8	8
gi|33859811	trifunctional enzyme subunit alpha, mitochondrial precursor [Mus musculus]	32	82617	3	3
gi|21703836	putative hexokinase HKDC1 [Mus musculus]	32	102193	2	2
gi|12843046	unnamed protein product [Mus musculus]	31	26390	19	2
gi|3378046	brush border myosin-I [Mus musculus]	31	104358	2	2
gi|74184761	unnamed protein product [Mus musculus]	30	128520	4	3
gi|29145087	Ogdh protein [Mus musculus]	29	116043	2	2
gi|74201981	unnamed protein product [Mus musculus]	29	59161	2	2
gi|7106255	arginase-1 [Mus musculus]	28	34786	2	2
gi|19526818	phosphate carrier protein, mitochondrial precursor [Mus musculus]	26	39606	2	2
gi|3273097	recA/RAD51 family protein [Mus musculus]	26	31381	5	3
gi|90855452	mKIAA0467 protein [Mus musculus]	25	167298	3	3
gi|148680468	mCG11265, isoform CRA_c [Mus musculus]	25	47195	2	2

gi|9055218	pre-mRNA-processing factor 40 homolog A [Mus musculus]	25	108412	2	2
gi|568932419	PREDICTED: uncharacterized protein C1orf167 homolog isoform X2 [Mus musculus]	23	85350	2	2
gi|74219810	unnamed protein product [Mus musculus]	22	94665	5	3
gi|26006239	mKIAA0998 protein [Mus musculus]	22	148193	2	2
gi|755516105	PREDICTED: glyceraldehyde-3-phosphate dehydrogenase isoform X1 [Mus musculus]	21	42250	5	3
gi|29470296	hydrocephalus-inducing protein [Mus musculus]	21	574705	8	6
gi|26327657	unnamed protein product [Mus musculus]	20	61698	2	2
gi|124487141	A disintegrin and metalloproteinase with thrombospondin motifs 3 isoform 2 precursor [Mus musculus]	19	135104	2	2
gi|118572948	RecName: Full=Histone-lysine N-methyltransferase NSD3 [Mus musculus]	19	160899	7	5
gi|1870416	anti-DNA immunoglobulin light chain IgG [Mus musculus]	18	12332	4	3
gi|21311939	cap-specific mRNA (nucleoside-2~-O-)-methyltransferase 1 [Mus musculus]	17	95615	3	2
gi|256665241	zonadhesin precursor [Mus musculus]	16	584262	5	4

**Band 4**					
gi|387397	epidermal keratin subunit I, partial [Mus musculus]	565	57807	24	7
gi|16303309	type II keratin 5 [Mus musculus]	400	61743	41	11
gi|741022	keratin 15 [Mus musculus]	270	49086	19	2
gi|398168	keratin 2 epidermis [Mus musculus]	214	70934	14	5
gi|7106335	keratin, type I cytoskeletal 17 [Mus musculus]	198	48132	17	2
gi|154090941	keratin, type I cytoskeletal 42 [Mus musculus]	137	50102	13	5
gi|148672085	mCG144996, partial [Mus musculus]	135	38119	12	3
gi|568992743	PREDICTED: keratin Kb40 isoform X1 [Mus musculus]	112	114116	6	3
gi|51092301	type II keratin Kb14 [Mus musculus]	77	60191	7	2
gi|568981521	PREDICTED: desmoplakin isoform X1 [Mus musculus]	67	261257	9	9
gi|809561	gamma-actin [Mus musculus]	46	40992	6	3
gi|51311	unnamed protein product [Mus musculus]	40	11445	2	2
gi|6996913	annexin A2 [Mus musculus]	33	38652	4	4
gi|700275391	anti-lox-1 15C4 heavy chain [Mus musculus]	32	51300	7	2
gi|21313536	dihydrolipoyllysine-residue succinyltransferase component of 2-oxoglutarate dehydrogenase complex, mitochondrial [Mus musculus]	30	48963	3	3
gi|97050032	RecName: Full=Filamin-A-interacting protein 1 [Mus musculus]	30	137513	3	2
gi|556310	spermatid-specific, partial [Mus musculus domesticus]	30	15099	2	2
gi|32129201	desmoglein-1-beta precursor [Mus musculus]	29	114382	3	3
gi|55408	vimentin [Mus musculus]	27	53689	4	2
gi|20071140	RIKEN cDNA 4933424B01 gene [Mus musculus]	18	82698	2	2

Inclusion of proteins: At least one top ranking peptide match and 2 unique peptides.
